# A system-view of *Bordetella pertussis* booster vaccine responses in adults primed with whole-cell versus acellular vaccine in infancy

**DOI:** 10.1172/jci.insight.141023

**Published:** 2021-04-08

**Authors:** Ricardo da Silva Antunes, Ferran Soldevila, Mikhail Pomaznoy, Mariana Babor, Jason Bennett, Yuan Tian, Natalie Khalil, Yu Qian, Aishwarya Mandava, Richard H. Scheuermann, Mario Cortese, Bali Pulendran, Christopher D. Petro, Adrienne P. Gilkes, Lisa A. Purcell, Alessandro Sette, Bjoern Peters

**Affiliations:** 1 Division of Vaccine Discovery, La Jolla Institute for Allergy and Immunology, La Jolla, California, USA.; 2 J. Craig Venter Institute, La Jolla, California, USA.; 3 University of California San Diego School of Medicine, La Jolla, California, USA.; 4 Department of Microbiology and Immunology, Stanford University School of Medicine, Stanford, California, USA.; 5 Regeneron Pharmaceuticals Inc., Tarrytown, New York, USA.

**Keywords:** Immunology, Vaccines, Bacterial vaccines, Imprinting

## Abstract

The increased incidence of whooping cough worldwide suggests that current vaccination against *Bordetella pertussis* infection has limitations in quality and duration of protection. The resurgence of infection has been linked to the introduction of acellular vaccines (aP), which have an improved safety profile compared with the previously used whole-cell (wP) vaccines. To determine immunological differences between aP and wP priming in infancy, we performed a systems approach of the immune response to booster vaccination. Transcriptomic, proteomic, cytometric, and serologic profiling revealed multiple shared immune responses with different kinetics across cohorts, including an increase of blood monocyte frequencies and strong antigen-specific IgG responses. Additionally, we found a prominent subset of aP-primed individuals (30%) with a strong differential signature, including higher levels of expression for *CCL3*, *NFKBIA*, and *ICAM1*. Contrary to the wP individuals, this subset displayed increased PT-specific IgE responses after boost and higher antigen-specific IgG4 and IgG3 antibodies against FHA and FIM2/3 at baseline and after boost. Overall, the results show that, while broad immune response patterns to Tdap boost overlap between aP- and wP-primed individuals, a subset of aP-primed individuals present a divergent response. These findings provide candidate targets to study the causes and correlates of waning immunity after aP vaccination.

## Introduction

A *Bordetella pertussis* vaccine containing heat-killed, whole-cell bacteria was developed in the first 2 decades of the 20th century, following the isolation of the organism in 1906 (1). After decades of research and improvements, the vaccine was put into routine use in the mid-20th century and has, since then, demonstrated its efficacy to confer immunity against the pathogen (1–4). For decades, this vaccine was administered in a mix with toxoid antigens derived from the causative agents of diphtheria and tetanus called DTwP. The pertussis compounds in this vaccine (“w” for whole-cell; also wP for short) were substituted during the 1990s by up to 5 individual protein antigens introduced in acellular vaccines for primary (DTaP) or booster (Tdap) vaccination (“a” for acellular; also aP for short) because of a significant reduction in side effects compared with DTwP. Although the DTaP/Tdap vaccine was shown to induce protection in infants (5, 6), questions were raised about its ability to induce long-lasting protection (7–9) and prevent transmission (10–12), as a number of countries recently experienced an alarming increase of whooping cough cases (13–15), leading to a concern that lack of efficacy of acellular vaccines might be the cause of recent outbreaks (16).

Several studies, including from our own group, characterized and compared immunological response in DTwP- and DTaP-primed individuals in terms of humoral (8, 17–19) and T cell–mediated immunity (20–24). These studies showed that both wP- and aP-primed individuals are capable of strong humoral responses, resulting in high levels of IgG against *Bordetella pertussis* antigens. However, T cell phenotype and polarization differed, and those differences persisted decades after the vaccine priming (25–27); this difference has been suggested to be linked with dissimilar vaccine efficacy and immune imprinting (27–30).

Attempts were also undertaken to compare immune responses in wP- and aP-primed individuals with Tdap booster vaccination (21, 22, 31–33). These reports were focused on the study and characterization of T cell or B cell memory responses and underlying differences between aP priming or wP priming. However, there has been no systematic assessment of all immune components accessible in blood, including innate and adaptive arms of the immune system and their interactions. Here, we sought to apply a diverse set of assays to probe the immune response development after vaccination at the systems level.

System immunology approaches have previously been applied to characterize vaccine responses against influenza (34), herpes virus (35), malaria (36), and others (37). We wanted to utilize this systems approach to characterize the response to Tdap boost of differently primed individuals. Vaccinated individuals were profiled on transcriptomic, proteomic, and blood cell composition levels during the first 2 weeks after booster vaccination, and humoral responses were profiled over a 3-month period. This set-up allowed us to uncover shared signatures marking the different stages of immune responses to Tdap boost, as well as subsets of individuals that showed divergent response patterns.

## Results

### Subject recruitment and study design.

**Figure 1:**
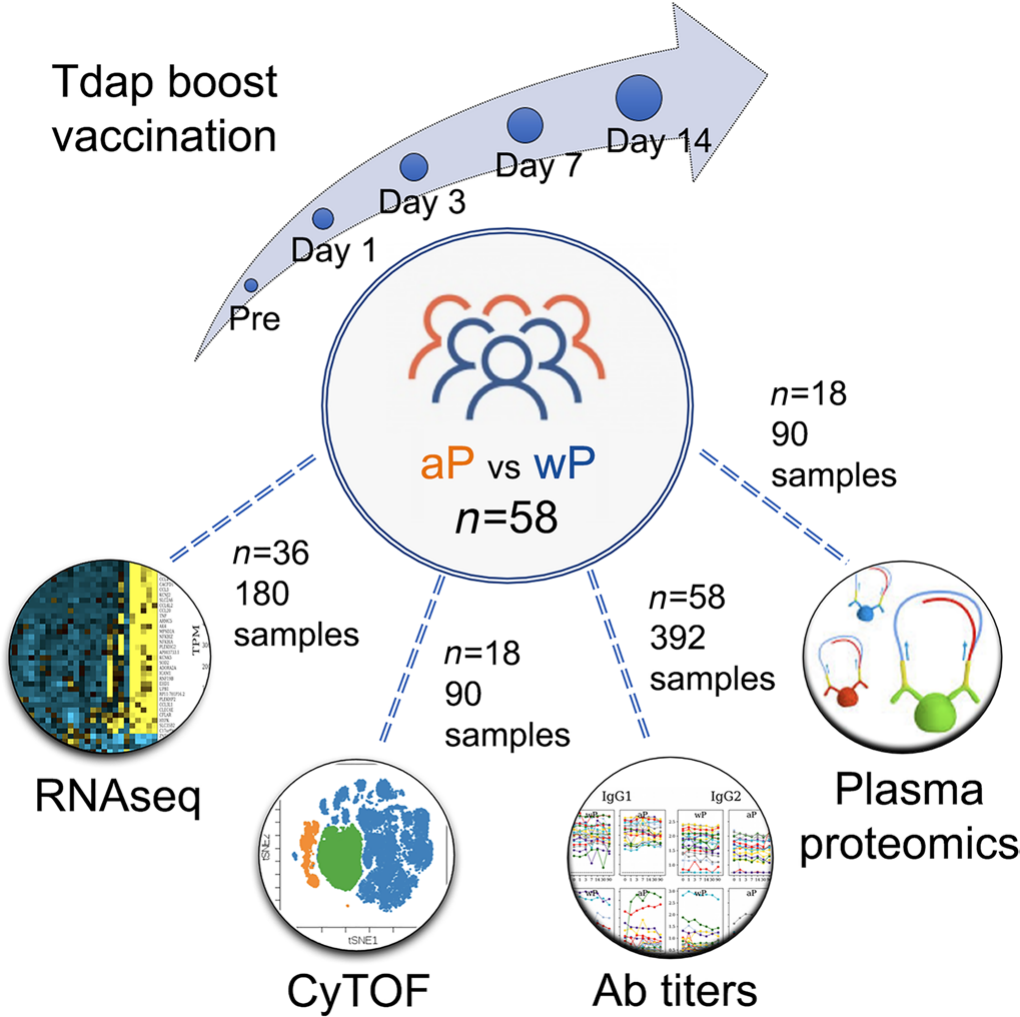
Outline of recruitment and study design. A total of *n* = 58 subjects were enrolled, and blood samples were collected prevaccination (day 0), and at days 1, 3, 7. and 14 following booster vaccination. In addition, plasma was collected at 1 and 3 months after vaccination. Four different sets of assays were performed: Gene expression by RNA-Seq in PBMC, protein marker expression by CyTOF, vaccine specific antibody titers by Luminex assay, and plasma protein concentration by PEA (proximity extension assay).

To study the long-term effect of priming with the aP versus wP vaccine, we recruited individuals born in the US prior to 1995, who will have been primed with the wP vaccine in infancy, versus individuals born later, who will have been primed with the aP vaccine. The recruited individuals were eligible for booster vaccinations with Tdap, containing tetanus toxoid (T), diphtheria toxoid (d), and acellular Pertussis (aP: filamentous hemagglutinin [FHA], fimbriae 2/3 [Fim2/3], pertactin [PRN], and inactivated pertussis toxin [PT]) antigens. We collected longitudinal blood samples prior to booster vaccination (day 0) and at days 1, 3, 7 and 14 after vaccination. Additional plasma samples were collected at 1- and 3-month postvaccination visits for the study of humoral responses on a subset of individuals who were available and for whom sample collection protocols were in place (Supplemental Table 1; supplemental material available online with this article; https://doi.org/10.1172/jci.insight.141023DS1). These samples allowed studying immune responses against *Bordetella pertussis* as a proxy to antigen encounter in vivo, and they allowed the study of whether this response differed in aP- versus wP-primed individuals 15 years or more after the original vaccination. To provide a system-level view of the vaccine-induced immune response, we set out to identify perturbations at the level of (a) gene expression in whole peripheral blood mononuclear cell (PBMC), (b) cell subset composition and activation states by mass cytometry (cytometry by TOF; CyTOF), (c) protein content in plasma, and (d) vaccine-specific antibody titers and isotypes in plasma. After dropouts, a total cohort of *n* = 58 donors was recruited, and different assays were performed on subsets of these donors, as summarized in Figure 1 and broken down per individual and assay in Supplemental Table 1. The number of donors recruited was chosen to be comfortably above our previous studies, which had a very similar recruitment strategy (21, 22) and which showed significant differences with a total cohort size of *n* = 33.

### Tdap boost induces perturbations in PBMC gene expression that follow distinct kinetic patterns.

**Figure 2:**
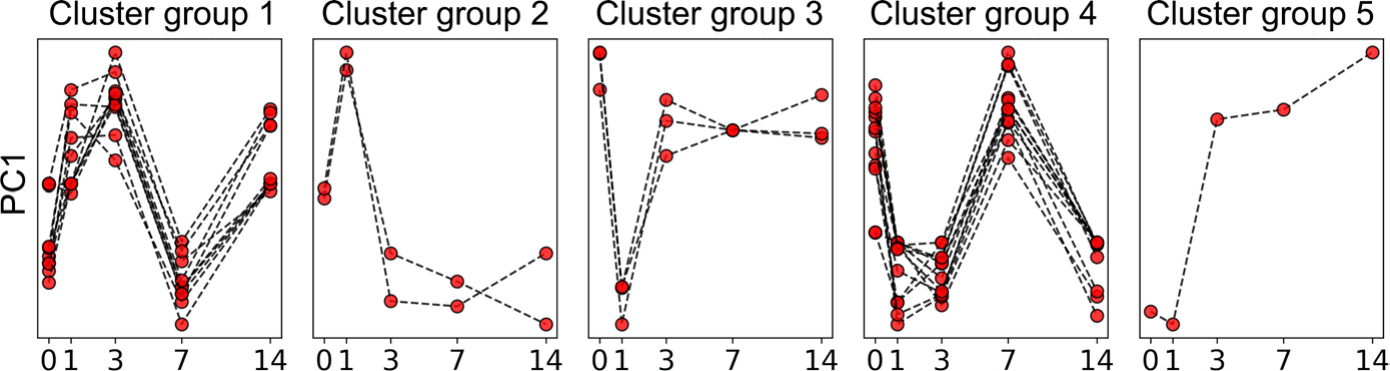
Kinetic groups of transcriptomic clusters. Every group consists of several of the 28 transcriptomic clusters evaluated in 36 donors across 5 time points (*n* = 36). Every line indicates minimum–maximum normalized PC1 of an individual cluster.

We performed RNA sequencing (RNA-Seq) analysis on PBMC samples collected longitudinally at baseline and following booster vaccination. A subset of 40 donors (20 aP versus 20 wP) for which the first complete time series was collected was selected for this assay. After RNA extraction and sequencing, samples that did not pass quality controls were removed (excluded for this assay only), and complete time-courses were obtained for *n* = 36 individuals (16 aP versus 20 wP). To reduce the dimensionality of the obtained gene expression data, we first performed an unbiased clustering analysis that grouped genes together that were coexpressed across samples from different time points and different donors. This identified 39 clusters of genes (annotated as TrC1…TrC39, Transcriptomic Clusters, Supplemental Data 1). For each sample, the expression level of genes in each cluster was quantified using a principal component analysis, where the first principal component (PC1) was taken as a proxy for expression of genes in the cluster. Inspecting the expression level of clusters at different time points after booster vaccination revealed that some clusters had essentially unchanged expression over time, while others showed clear perturbations (Supplemental Figure 1). To quantify which clusters were significantly perturbed by the booster vaccination, we picked the time points of highest and lowest expression for each cluster, and we then determined if the difference in expression between these 2 points was statistically significant using the paired, nonparametric Wilcoxon test (Supplemental Table 2). We performed this comparison using data from (a) all individuals, (b) only aP-primed individuals, and (c) only wP-primed individuals. Agglomerative cluster analysis was further performed, and 5 groups of clusters were generated, taking into consideration the hierarchical relationship of the patterns of the individual transcriptomic clusters as shown in the dendrogram of Supplemental Figure 2 and described in more detail in the Methods. Overall, this identified that 28 of the 39 gene clusters showed significant perturbations after vaccination in at least 1 of the 3 comparisons made. Remarkably, only 1 cluster (TrC21; cluster group 5) out of 28 showed perturbations that were limited to 1 comparison, namely to wP-primed individuals; all other clusters showed either consistent perturbations in all 3 comparisons or none at all. The 28 perturbed gene clusters and associated expression kinetics are shown in Figure 2. The first group consisted of 10 clusters (2346 genes in total) and was characterized by an early increase after vaccination at day 1 and 3. Group 2 consisted of 2 clusters (246 genes in total) with a distinctive early peak of expression at day 1. Group 3 consisted of 3 clusters (262 genes in total) that had significant drops in expression at day 1, which recovered nearly to baseline at day 3. Group 4 included 12 clusters with a substantial peak at day 7 that comprised 2360 genes in total. Group 5 consisted of a single cluster showed increases in expression starting at day 3. Overall, Tdap booster vaccination induced significant perturbations in gene expression of PBMC as detected by RNA-Seq, and these perturbations followed distinct kinetic patterns.

### Tdap boost induces changes in PBMC cell composition that correlate with changes in gene expression.

**Figure 3:**
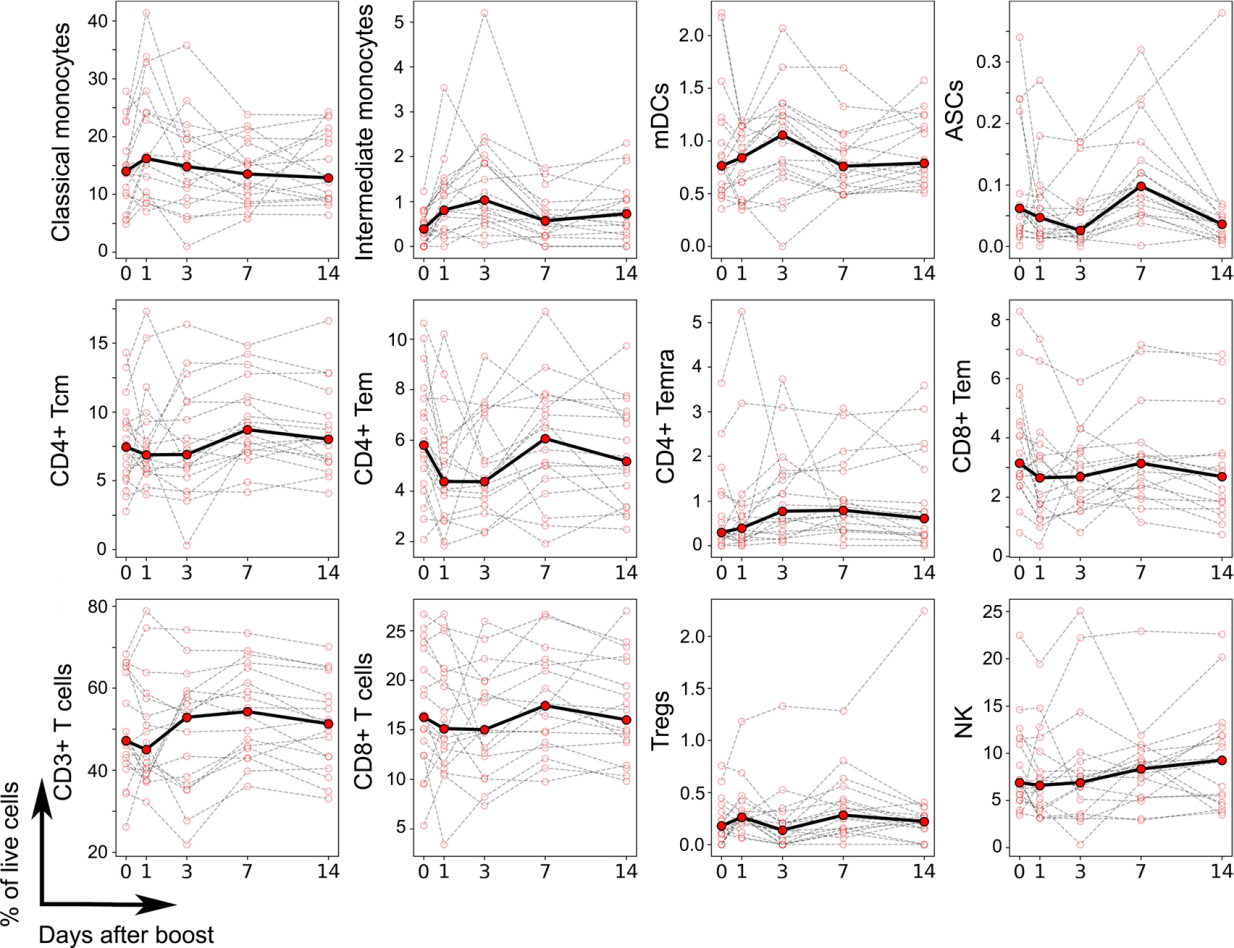
Frequency of cell types altered by vaccination as determined by CyTOF. Each plot represents the frequency of a given cell type in terms of percentage of live PBMC (*y* axis) as a function of the time after booster vaccination (*x* axis), as determined by high-dimensional automated gated analysis of data generated by CyTOF. Data are expressed as connected time points from day 0 to days 1, 3, 7, and 14 after booster vaccination for each individual donor (thin lines) and the median of all (bold line). A total of 18 participants was evaluated (*n* = 18), and 21 cell types were evaluated. Only cell types that showed a significant difference in frequencies over time are shown, based on a paired, nonparametric Wilcoxon test (*P* < 0.05).

PBMC compose a heterogeneous mixture of different cell types that vary substantially in frequency between individuals even in the absence of immune perturbations (38). Differences in cell type frequencies in PBMC across individuals are expected to impact gene expression patterns. We systematically analyzed variations in cell type frequencies across different time points for *n* = 18 donors (8 aP- versus 10 wP-primed donors) using a CyTOF panel (see Supplemental Figure 3 for gating strategy and Supplemental Table 3 for phenotype). High-dimensional automated gating analysis using the Directed Automated Filtering and Identification of cell populations (DAFi) method (39) allowed identification of 21 predefined/distinct cell types and their frequencies, including cell populations that are difficult to resolve by manual gating analysis such as memory T cells and Tregs (Supplemental Figure 4). We determined which of these cell types showed significant perturbations after boost in all individuals, or for either aP- or wP-primed individuals separately. This identified a total of 12 cell populations that showed perturbations (Figure 3) — including myeloid cells (classical and intermediate monocytes, myeloid DCs [mDCs]), which increased at days 1–3 after boost, and antibody-secreting cells (ASCs), which peaked at day 7. NK cells were characterized by a steady increase through the 14 days of observation. Most of the T cell subsets with significant perturbations showed a decrease at day 1, day 3, or both (CD4^+^ central memory T cell [Tcm], CD4^+^ effector memory T cell [Tem], CD8^+^ Tem, CD3^+^ T cells, CD8^+^ T cells), while 2 T cell populations (CD4^+^ effector memory T cells reexpressing CD45RA [Temra] and Tregs) instead showed a slight increase at day 1 and/or day 3. Overall, similar to the gene expression data, we found a heterogeneous set of kinetics upon Tdap boost associated with different cell types.

**Figure 4:**
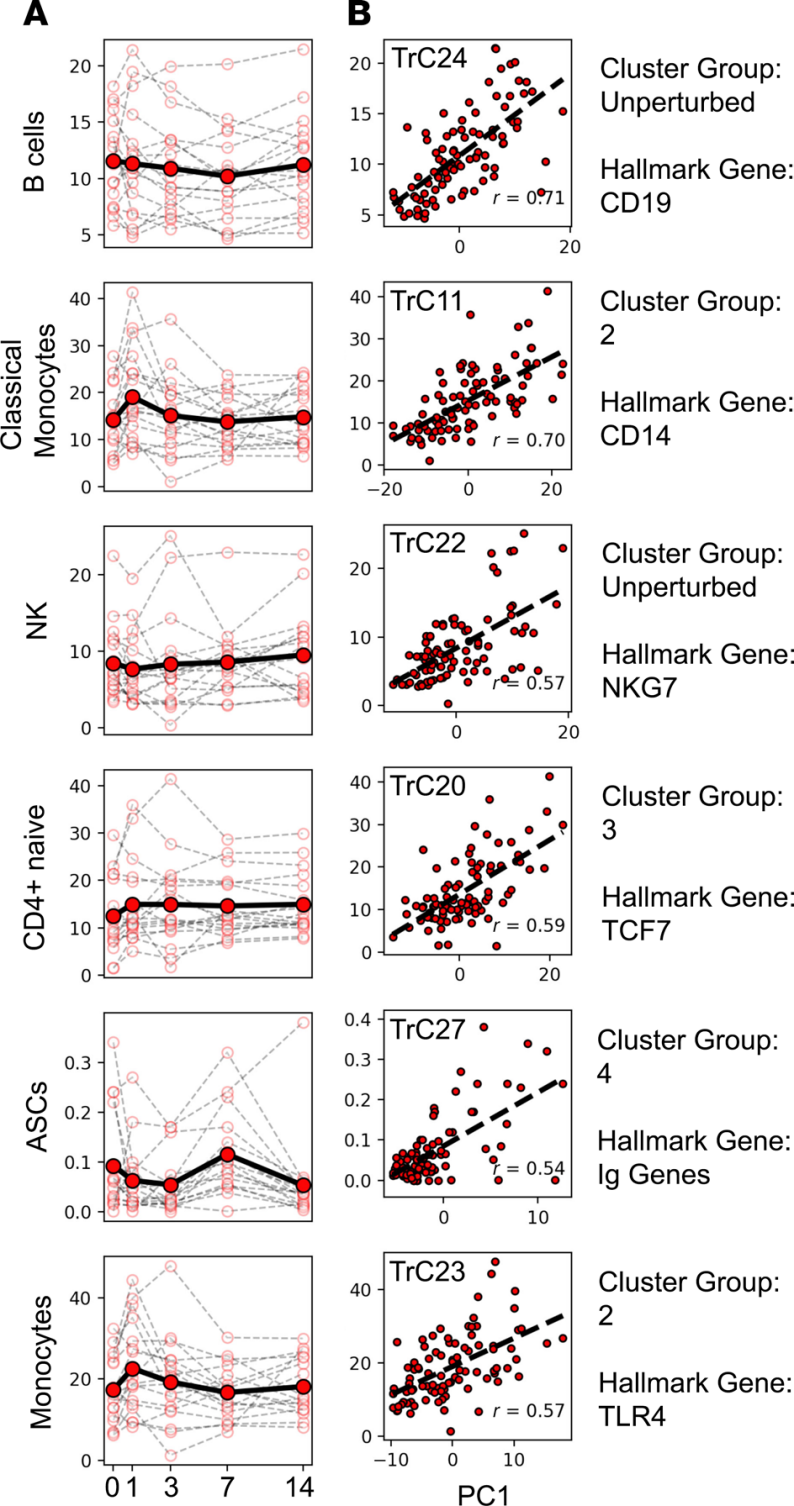
Frequencies of several cell types correlate highly with gene expression levels of specific clusters. The plots show all cell types for which Spearman’s correlations of *r* > 0.5 were found with a specific gene cluster — namely B cells, classical monocytes, NK cells, CD4 naive cells, ASCs, and monocytes (*n* = 90 equivalent to 18 individuals × 5 time points). (**A**) Frequency of the cell type percentages of live cells are plotted as a function of time post booster vaccination. Open circles and thin lines connecting them indicate individual responses. Closed circles and the solid lines connecting them indicate average responses over all donors. (**B**) Correlation of cell type frequencies with gene expression of the best-matching RNA-Seq cluster (different cluster for every cell type), quantified by the first principal component (PC1). The specific cluster is indicated on the top left corner of each plot, while its cluster group and a hallmark gene are indicated on the right.

Next, we correlated the frequency of cell types determined by CyTOF with the expression level of gene clusters identified by RNA-Seq for *n* = 90 samples (18 subjects × 5 time points) where both RNA-Seq and CyTOF data were available (Supplemental Table 4). We found several gene clusters whose expression was highly correlated with cell type frequencies (Figure 4). This included cluster TrC24, which correlated (*r* = 0.71) with frequency of B cells across samples. TrC24 contains the B cell lineage marker CD19, further suggesting that its expression is directly linked to the frequency of B cells in each sample. Notably, the gene cluster shows no significant perturbation in average expression over time after boost — and neither does the B cell frequency. Thus, the different expression levels of genes in the TrC24 cluster reflect different baseline frequencies of B cells across individuals, ranging from 5% to 20% of PBMC, which were not systematically impacted by the booster vaccine. In contrast to B cells overall, cluster TrC27 was significantly correlated with the frequency of ASCs (*r* = 0.58). Of the 49 genes in TrC27, 38 code for Ig heavy and light chains. Both expression of this cluster and frequency of ASCs peak at day 7, which is in line with previous reports of a peak in antibody production at day 7 after booster vaccination in other systems such as influenza (34, 40). Thus, Tdap booster vaccination does not perturb the overall level of B cells in PBMC, but it does increase the frequency of ASCs, which peak at day 7, corresponding with the kinetics of the group 4 gene cluster.

The second highest correlation for gene cluster expression and cell type frequency was observed for classical monocytes and the gene cluster TrC11 (*r* = 0.70, Figure 4). The TrC11 cluster contains *CD14*, the lineage marker of classical monocytes, and falls into cluster group 2, which peaked in expression at day 1 after vaccination. This cluster also contained the *LYZ* and the *S100A9* genes, which distinguish classical from nonclassical monocytes. The parent population of total monocytes also correlated significantly with the group 2 transcriptomic cluster TrC23, which contains genes shared in both classical and nonclassical monocytes such as *TLR4*. Overall, these data suggest that the peak of expression of multiple genes at day 1 after booster vaccination resulted from an increase in the frequency of monocytes in PBMC, of which classical monocytes make up the vast majority.

The next 2 cell types that correlated highly with specific gene expression clusters were NK cells, which showed high correlation with TrC22, and naive CD4^+^ T cells. While the perturbation in NK cell frequency was significant, the perturbation in expression of TrC22 was below the threshold of significance, which resulted in it being classified as unperturbed. The gene cluster did contain *NKG7*, though, which is an NK signature gene; therefore, the correlation is still likely to be biologically meaningful. Naive CD4^+^ T cell frequency correlated strongly with TrC20. While the perturbation of the cell frequency was not significant, the perturbation of the gene expression cluster did reach significance. The cluster contained *TCF7*, a hallmark gene of CD4^+^ T cells. As for NK cells above, this suggests that the correlation between these cell frequencies and the gene cluster are likely to be biologically meaningful.

### Tdap boost elevates selected cytokine concentrations in plasma.

To determine how Tdap vaccination impacts proteins secreted into blood, we employed a proteomics approach to uncover vaccination-related perturbations of 241 unique plasma protein markers. A total of 80 plasma samples (16 subjects × 5 time points) was profiled (6 aP- versus 10 wP-primed donors) and evaluated for expression of proteins using a highly sensitive and quantitative proximity extension assay (PEA) (41). In order to study the kinetics of cytokines and other factors in plasma, we performed a dimensionality reduction approach similar to what we performed for gene RNA expression (Supplemental Figure 5) and obtained 8 protein clusters of 5 or more proteins (Supplemental Figure 6) with 209 proteins that presented perturbation across the study (Supplemental Data 2).

We then examined if any of these clusters was significantly perturbed over time in all individuals or only in aP- or wP-primed individuals. We found that only 1 cluster (PrC07) was significantly perturbed, which contained proteins encoded by *MASP1*, *TNFSF14*, *CCL3*, *CCL4*, *HGF*, *CLEC6A*, *CXCL8*, *CLEC4D*, and CLEC4C. This cluster showed significant increased protein levels at days 1 and 3 after booster vaccination and a return to baseline levels afterward. When comparing the expression of the PrC07 cluster with gene transcriptomics and cell type frequencies, we found that PrC07 positively correlated with 3 transcriptomic clusters (TrC09, TrC10, and TrC31) and negatively correlated with 7 clusters (TrC03, TrC5, TrC7, TrC13, TrC28, TrC34, and TrC39), all with an absolute *R* > 0.5. All the positively correlated transcriptomic clusters belonged to cluster group 1 (peaking at days 1 and 3), and 6 of 7 negatively correlated clusters belonged to cluster group 4 (upregulated at day 7). Overall, this showed that some protein concentrations in plasma had a distinct and transitory kinetic response to booster vaccination.

### Tdap boost induces an isotype- and antigen-specific pattern of antibody production, with transcriptomic changes preceding increased antibody titers.

**Figure 5:**
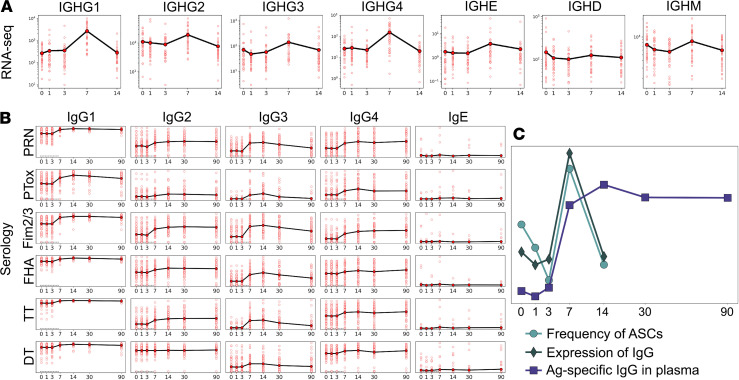
Longitudinal patterns of humoral response. In all plots, the *x* axis denotes days after vaccination. Averaging is done within all cohorts. (**A**) Expression of Ig constant regions from RNA-Seq for a total of 36 donors (*n* = 36). (**B**) Tdap-specific IgG subclass titers (log_10_-scaled) from plasma expressed in the mean of 58 donors (*n* = 58). (**C**) Overlay of values from RNA-Seq (*n* = 36), antibodies (*n* = 58), and CyTOF (*n* = 18) combined measurements. Average of PC1 across all the profiled donors is plotted for RNA-Seq and IgG titers data, as well as the cell frequency specific for ASCs.

To characterize the humoral response to Tdap, we first profiled the expression of Ig genes in PBMC transcriptomic data. RNA-Seq reads were mapped to the constant regions encoded by *IGHG1–4*, *IGHE*, *IGHM*, and *IGHD* genes, followed by transcripts per million reads (TPM) transformation. As shown in Figure 5A, RNA expression of all IgG heavy chains peaked at day 7, and a similar pattern was observed for IgM. Transcripts of the IgD heavy chain, on the contrary, did not demonstrate a profound increase at day 7, and the increase in IgE transcripts showed a smaller, insignificant, increase.

We next profiled the plasma levels of antigen-specific IgG, all IgG subclasses (IgG1–4), and IgE in the same kinetic fashion as before (1–14 days) and up to 3 months after boost. Igs specific for the antigens contained in the Tdap vaccine were assessed, namely PT (1% paraformaldehyde [PFA] and Pertussis toxin mutant [PTM] each), PRN, FHA, FIM2/3, tetanus toxoid (T) and diphtheria toxoid (d) (Supplemental Figure 7). As a control, 2 whole-cell vaccine antigens not contained in Tdap (adenylate cyclase and lipopolysaccharide of *Bordetella pertussis*) were also profiled, and as expected, no increase of wP-specific IgG levels was observed after booster vaccination (Supplemental Figure 8). In contrast, we observed strong increases in all IgG subclass levels against antigens contained in Tdap (Figure 5B) starting at day 7 of the observation period. The peak of antigen-specific IgG levels was observed on day 14, whereas by day 30, the Ig levels started to decrease. Interestingly, IgG3 levels decreased the fastest (Figure 5B). Tdap-specific IgG antibodies were induced in an antigen- and IgG subclass–specific fashion. For example, there was almost no IgG2 response against diphtheria toxoid (d) (Figure 5B). The strongest responses were observed against FIM2/3 antigen (especially IgG1 and IgG3). Overall, when averaging ranks across antigens, the IgG1 response was the strongest, while IgG2 was the weakest. In concordance with Ig RNA levels, there was no consistent induction of IgE expression.

An overlay of 3 signals related to antibody production is depicted in Figure 5C — namely, the detection of antigen-specific IgG in plasma; the RNA expression levels of IgG, IgE, IgD, and IgM genes in PBMC; and the frequency of ASCs in PBMC by CyTOF. Figure 5C indicates that production of Ig peaks around day 7, as assessed by gene expression and frequency of ASCs. The level of antigen-specific antibodies in plasma peaks at day 14 and stays elevated until day 90, which is consistent with antibodies having a half-life of several weeks (42). Overall, the data provide consistent evidence for an increased influx of antibody-producing cells into PBMC peaking at day 7 after booster vaccination, which results in a persistent increase in *Bordetella pertussis*–specific circulating antibodies.

### Differences in the cellular immune response to Tdap boost in wP- versus aP-primed individuals.

After characterizing common patterns of immune responses following Tdap booster vaccination, we wanted to identify differences in the response of aP- versus wP-primed individuals. First, we applied Mann-Whitney *U* test to determine if the expression level of any transcriptomics gene module differed significantly between aP and wP individuals at different time points (Supplemental Table 5). While most of the 31 gene clusters showed no significant difference in expression between wP- and aP-primed individuals at any time point, the 3 clusters that did show significantly different expression at specific time points were TrC27, TrC31, and TrC38. As stated above, the TrC27 cluster correlated strongly with the frequency of ASCs, with a slow reduction in expression or cell frequency at days 1 and 3 after boost and peaking at day 7 after boost, regardless of the priming vaccine composition (aP or wP). In parallel, we also evaluated group differences for the frequency of ASCs, and although higher average frequencies were observed in the aP-primed cohort at all the time points, this did not reach statistical significance (Supplemental Figure 9). Concordantly, the gene cluster TrC27 also showed higher expression in aP versus wP individuals at day 0, prior to booster vaccination. To determine if the higher baseline expression of TrC27 in aP donors was correlated with baseline antibodies, we correlated titers of the different antibody measures with expression of TrC27, for which data were available. While the majority of antibody measures did not correlate, a significant correlation was observed for anti-LOLP11 IgG1, anti-PRN IgG3, and anti-FIM IgG3 (Supplemental Figure 10A). Out of the correlated antibody measures, anti-LOLP1 IgG1 and anti-FIM2/3 IgG3 were higher in aP donors at the baseline (Supplemental Figure 10B) and might be indicative of antigen encounter that recently triggered ASC responses. In conclusion, although the baseline level differences could not be explained with our experimental design, the perturbation observed in TrC27 and in the frequencies of ASCs is induced by Tdap booster vaccination.

**Figure 6:**
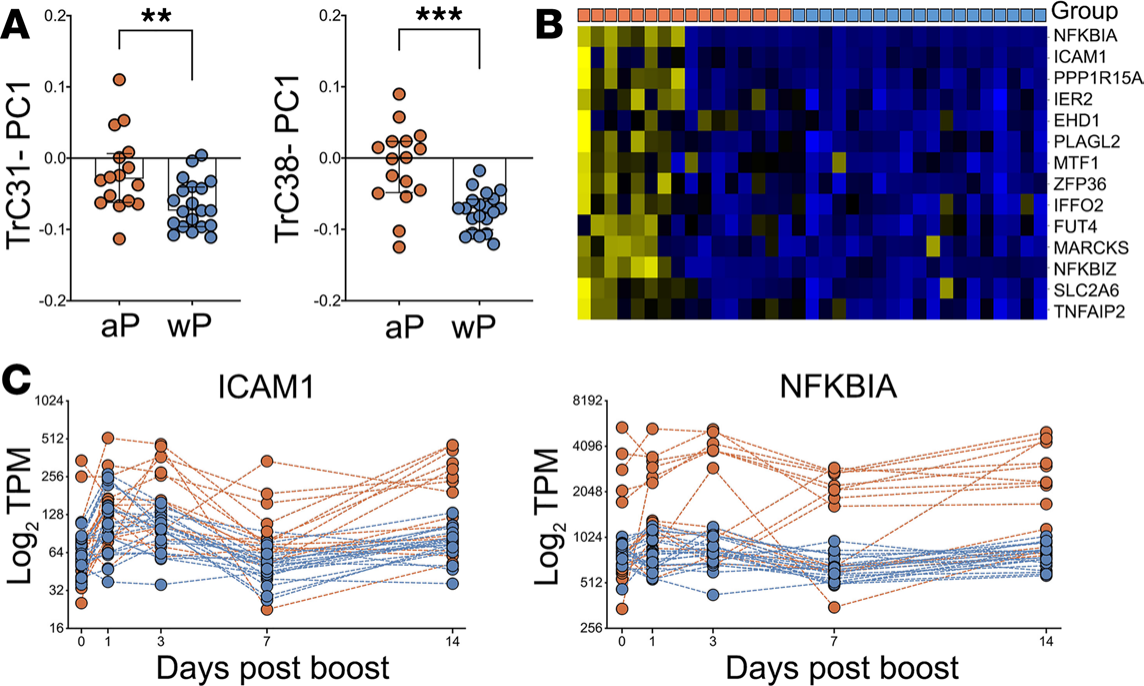
Gene expression differences in aP versus wP individuals on day 7. (**A**) Expression of the genes in clusters TrC31 and TrC38 is significantly different 7 days after boost in aP versus wP individuals (*n* = 16 for aP and *n* = 20 for wP) as quantified by the principal component analysis (PC1). Statistical differences were evaluated using a 2-sided, nonparametric Mann-Whitney *U* test (**P = 0.006, ***P = 0.0002). Median and IQR are represented by box and error bars, respectively. (**B**) Heatmap of 14 genes from clusters Trc31 and -38 that were identified as differentially expressed on day 7 (Benjamini-Hochberg *P*_adj_ < 0.05). Columns denote group of individuals originally primed with either aP or wP as indicated by orange versus blue boxes in the top row (*n* = 16 for aP and *n* = 20 for wP). (**C**) Expression level of *ICAM1* and *NKBIA* over time as representative genes in clusters TrC31 and TrC38, respectively, that are differentially expressed on day 7 between aP and wP individuals (*n* = 16 for aP and *n* = 20 for wP).

Clusters TrC31 and TrC38 showed higher expression in aP- versus wP-primed individuals at day 7 after boost (Figure 6A). To determine what specific genes in these clusters contribute to the difference in aP- versus wP-primed individuals at day 7, we performed differential expression analysis using DESEQ2, limited to the genes in these 2 clusters. This identified a total of 14 genes with *P*_adj_ < 0.05: 4 from TrC31, the most pronounced of which was *ICAM1*, and 10 from TrC38, with the most pronounced being *NFKBIA*. Figure 6B shows a heatmap of the expression of these 14 genes in the overall cohort at day 7. This highlights that the differences between aP and wP individuals were due to a subset of aP individuals who showed high expression of these genes compared with any of the wP donors. These results also suggest that intracohort variability in aP- but not wP-primed individuals were driving the differences we detected here.

**Figure 7:**
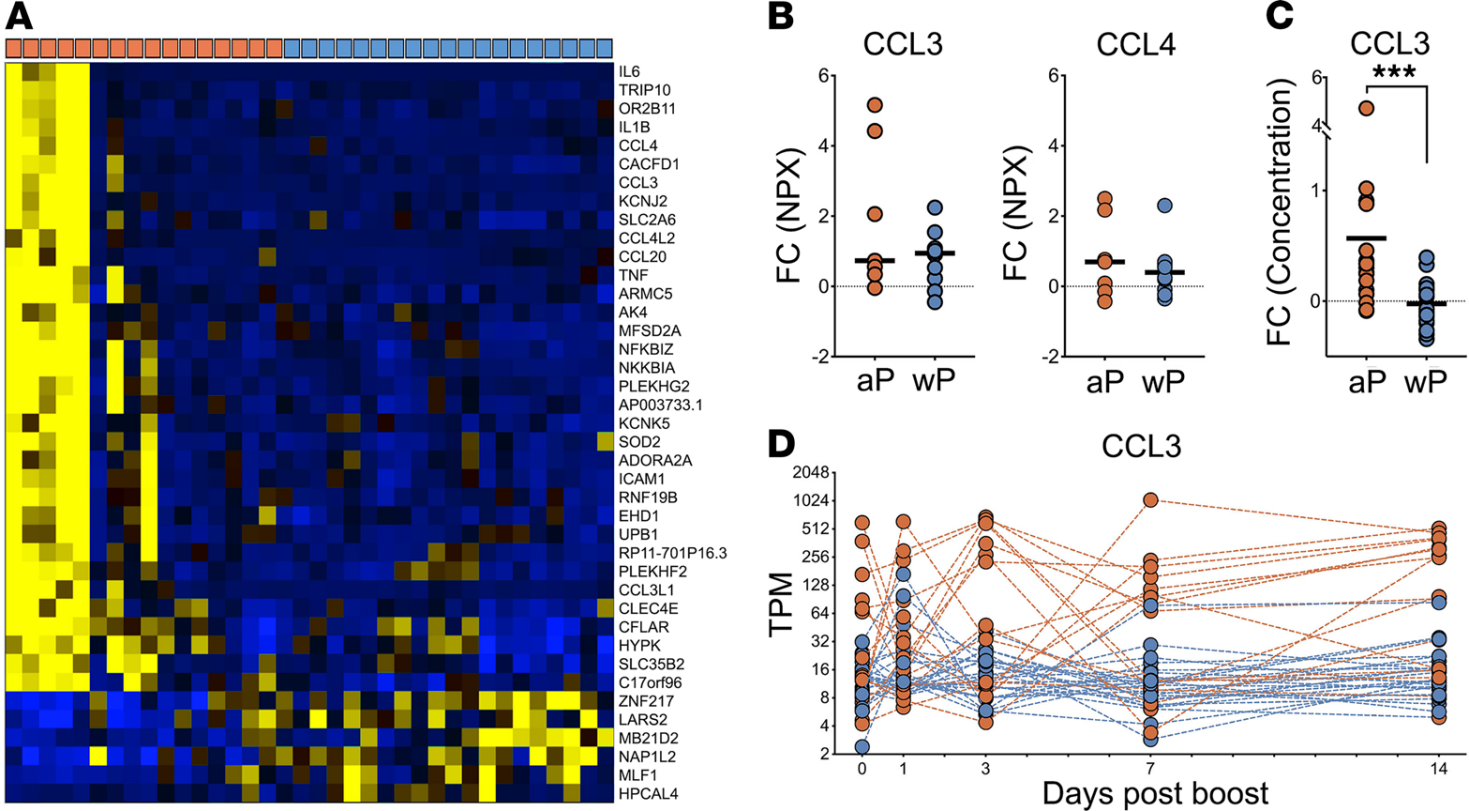
Observed differences in aP and wP-primed individuals on day 3. (**A**) Heatmap of genes differentially expressed between the cohorts on day 3. Columns denote different individuals primed with aP versus wP, as indicated by orange or blue boxes, respectively, in the top row (*n* = 16 for aP and *n* = 20 for wP). (**B**) Differences in plasma concentration of CCL3 and CCL4 between day 0 and day 3 based on change of NPX values as obtained by PEA assay in human plasma (*n* = 6 for aP and *n* = 10 for wP). (**C**) Log_2_ fold change from preboost to day 3 of plasma concentrations of CCL3 by ELISA. Statistical differences were evaluated using a 2-sided, nonparametric Mann-Whitney *U* test (***P = 0.0002). (**D**) Modulation of *CCL3* gene expression over time. aP and wP donors are represented in orange and blue, respectively (*n* = 16 for aP and *n* = 20 for wP).

Examining the kinetics of *ICAM1* and *NFKBIA* as representatives of differentially expressed genes in cluster TrC31 and TrC38 (Figure 6C) revealed that the subset of aP-primed individuals that showed enhanced expression of these genes at day 7 also had high expression at other time points. Specifically, 3 of the 4 individuals with the highest *ICAM1* expression at day 7 had even higher *ICAM1* expression at day 3, and 6 of the 9 aP individuals with higher *NFKBIA* expression than any wP individual on day 7 already had higher expression than wP individuals on day 3. To examine this in more detail, we performed differential gene expression analysis between aP- and wP-primed individuals at day 3 after boost. Thirty-six genes were identified as differentially expressed with *P*_adj_ < 0.05 between these cohorts, independent of any specific module considered (Figure 7A). As expected, aP-primed individuals again had significantly higher expression of *NFKBIA* and *ICAM1* at day 3. In addition, inflammatory cytokines such as *CCL3*, *CCL4*, *IL6*, and *TNF* were also expressed highly in the same subset of aP individuals. The identified signature of 36 genes that distinguish this subset of aP donors included several gene ontology (GO) terms associated with inflammation and chemotaxis of immune cell subsets (Supplemental Figure 11A). Specifically, the top 5 terms were: inflammatory response, monocyte chemotaxis, cytokine-mediated pathway, cellular response to IL-1, and regulation of ERK1 and ERK2 cascade. Nine genes of this set recurred across these pathways: *CCL3*, *CCL4*, *CCL20*, *CCL3L1*, *IL1B*, *IL6*, *TNF*, *NFKBIA*, and *ICAM1*. These genes are thought to be mainly produced by monocytes, NK cells, and T cells (Supplemental Figure 11B), suggesting that the observed differences are at the interface of innate and adaptive immunity. Differences in cell type frequencies in PBMC across aP- versus wP-primed individuals were also analyzed, but we did not detect significant group differences between cohorts, and we did not have sufficient samples to compare intracohort variability at individual time points.

To examine if the increased transcription of the cytokine genes was also reflected in increased protein expression, we examined the concentration levels of CCL3 and CCL4 in plasma as resulted from PEA. Indeed, we found increased expression in aP compared with wP individuals (Mann-Whitney *U* test, *P* = 0.012), and this trend was also obtained for CCL4 (Figure 7B). To increase the number of individuals for whom we had data available, we analyzed CCL3 concentrations using ELISA in a broader set of plasma samples (38 donors = 17 wP versus 21 aP), which confirmed that the aP-primed individuals had significantly (*P* < 0.0001, Mann-Whitney *U* test) higher induction of CCL3 in plasma on day 3 than wP-primed individuals (Figure 7C). This confirmed that a subpopulation of aP individuals showed a distinct inflammatory cytokine production on day 3. Interestingly, the same subset of aP-primed individuals with high expression of the CCL3 gene at day 3 also had high expression at other time points (Figure 7D).

### Differences in the humoral immune response to Tdap boost in wP- versus aP-primed individuals.

**Figure 8:**
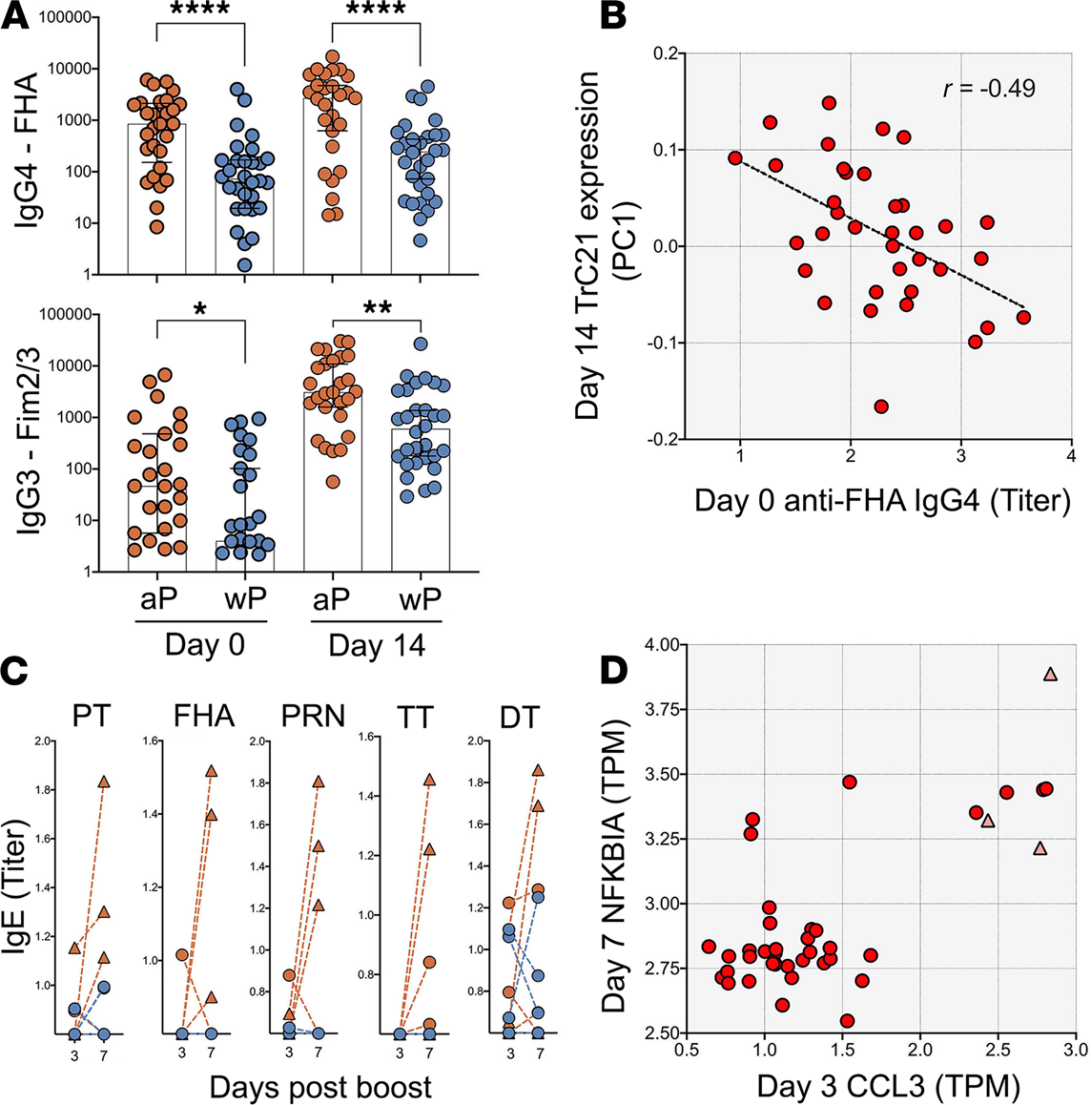
Differences in humoral responses against the pertussis vaccine antigens in aP versus wP donors. (**A**) FHA and Fim2/3 prior to booster vaccination and 14 days after. Statistical differences between aP and wP primed individuals (*n* = 28 for aP and *n* = 30 for wP) were evaluated using a 2-sided, nonparametric Mann-Whitney *U* test (****P < 0.0001, **P = 0.0015, *P = 0.0141). Geometric mean and SD are represented by error bars. (**B**) IgG4 antibody titers prior to boosting (*x* axis) negatively correlate with the expression of module TrC21 14 days after boost. Correlation was evaluated using Spearman’s correlation test (*r* = –0.49) (*n* = 36). (**C**) IgE level modulation over time for the several antigens in individuals vaccinated with aP (red) or wP (blue lines) in infancy (*n* = 28 for aP and *n* = 30 for wP). Three individuals from the aP group (triangles) showed consistent increases of IgE from day 3 to day 7 after booster vaccination. (**D**) Scatter plot of RNA-Seq gene expression data of *NFKBIA* on day 7 (*y* axis) versus *CCL3* expression on day 3 (*x* axis). The 3 individuals with consistent IgE responses are marked with red triangles (*n* = 36).

We further interrogated whether differences in antibody responses between aP- and wP-primed individuals could be identified by looking at the antibody isotypes induced by booster vaccination against the specific antigens contained in the Tdap vaccine. We previously defined that Tdap-specific antibodies were induced by day 7 after booster vaccination. In comparing the total IgG titers against several Tdap antigens between aP (*n* = 28) and wP (*n* = 30) cohorts (Supplemental Figure 7), no significant differences were found at this time point. Next, we looked into antigen-specific response at the level of IgG subclasses. After FDR correction, we found that, at the peak of the antibody boost on day 14, aP-primed individuals showed higher IgG4 response against FHA and higher IgG3 response against FIM2/3 than wP-primed individuals. Importantly, these higher titers in aP individuals were already observed prior to the booster vaccine, suggesting long-lived differences in the level of FHA-specific antibodies of these isotypes between the cohort (Figure 8A). To evaluate if the baseline differences in anti-FHA IgG4 between aP and wP was vaccine specific, we evaluated the levels of IgG3 and IgG4 against 3 nonvaccine antigens — OVA, FELD1, and LOLP1 (Supplemental Figure 12) —and no significant differences were observed. Since the prebooster titers for the IgG4 subclass antibodies in particular were substantial, we examined whether the difference in IgG4 antibodies prior to boost correlated with any of our measures of the cellular immune response at later time points. The highest correlation we obtained was *r* = –0.49 for cluster TrC21 (Figure 8B), which was the sole cluster falling into the kinetic group 5 that peaked on day 14 and was only found significantly perturbed in wP-primed individuals. This cluster contained genes such as the chemokine CXCL5 and several integrins associated with platelets.

In light of the observations linking antibody baseline levels and gene expression, we evaluated the possibility that the subset of aP donors with high expression of proinflammatory genes at 3 and 7 days after boost had been recently exposed to *Bordetella pertussis* by evaluating baseline antibody titers against a *Bordetella pertussis* antigen not contained in the vaccine. Surprisingly, although the number of donors for comparison was small for this subset of aP donors, it presented significantly higher levels of anti–ACT IgE, a trend to higher levels of anti–ACT IgG4 with respect to wP-primed individuals, and similar levels of any other IgG isotype (Supplemental Figure 13).

We further analyzed antigen-specific IgE responses, which have previously been reported as being enhanced in aP individuals (18, 43, 44) — especially with underlying atopic conditions (45). The majority of subjects did not demonstrate any IgE increase at day 7, but 3 out of 30 aP-primed individuals (10%) did possess a significant increase in IgE (at least by 50%) levels against 3 or more Tdap antigens (Figure 8C). For wP-primed individuals, IgE responses were not consistent across antigens: 2 donors responded with IgE against diphtheria toxoid (d) only, and 1 donor against tetanus toxoid (T) (nonpertussis antigens). As a control for allergic status of the donors, we also looked at IgE response against 2 seasonal allergens. We observed 2 potentially allergic donors in the aP cohort and 2 potentially allergic donors in the wP cohort, and none of those donors were the aP donors presenting high vaccine-specific IgE levels at 7 days after boost. Although it could not be confirmed by clinical records, these results suggested that the tendency for a higher IgE response in aP individuals was not driven by an increased presence of allergic individuals. Notably, while the number of individuals with increased Tdap-specific IgE response was small, all 3 of these individuals were also among the high *CCL3* expressers on day 3 and high *NFKBIA* expressers on day 7 (Figure 8D).

Overall, these results suggest that the differential gene and protein expression patterns observed in a subset of aP individuals might be linked with differential antibody isotype polarization, and it might be caused by asymptomatic exposure to *Bordetella pertussis*.

## Discussion

In this study, we aimed to utilize multisystem profiling of immunological response to Tdap booster vaccination in wP- and aP-primed individuals to uncover immune signatures of the booster immunization that are shared across individuals, as well as those that differ based on the type of the priming vaccine.

In terms of shared immune signatures, we found significant alterations in the transcriptomic profile on days 1 and 3 after boost, reflecting innate and early onset of adaptive immunity. Several of the observed changes could be explained by changes in the cell composition in PBMC, which showed a marked increase in the frequency of monocytes on day 1 and 3, and an accompanying decrease of other cell types. Of note, given that RNA-Seq data measure relative concentrations of gene expression in PBMC, and given that the cell frequency data we obtained represent a proportion of total live cells, it is possible that most of the changes observed are explained by an absolute increase in the release of monocytes into the bloodstream following vaccination, which has been reported before (46). Such an absolute increase of monocytes would also result in a decrease of the relative frequency of other cell types, such as T cells.

Beyond the vaccine-induced alterations in transcription on days 1 and 3 that could be explained by cell type composition of PBMC, we also found transcriptomic perturbations that were indicative of cell activation such as in the case of *CD14*, *TLR4*, or *S100A* gene expression. It was recently shown that CD14 is a coreceptor of TLR4 in the S100A9-induced cytokine response and is involved in the activation and proinflammatory response of monocytes (47). Several of these changes were accompanied by a steady increase in detection of proteins in plasma, including *CCL3*, TNFSF14, *CXCL8*, and several C-type lectins. CCL3 is a proinflammatory cytokine affecting many immune cells (48). It is also known to induce a Th1 response and affect T cell differentiation (49). Tumor necrosis factor superfamily ligand TNFSF14 (also known as LIGHT) is a regulatory molecule that can be produced in membrane-bound as well as soluble form. It can affect inflammatory as well as structural cells (50, 51). It can costimulate T cell activation and proliferation (52). CXCL8 (IL-8) is another proinflammatory cytokine involved in neutrophil recruitment (53). Induction of CXCL8 secretion by PMBC was observed in a study of the shingles vaccine (35). CCL3 and TNFSF14 were not reported in that or other studies of antiviral vaccines (34, 36) and potentially highlight a specific response to Tdap booster vaccination.

On day 7 after boost, we observed significant perturbations across our readouts that were consistent with a peak of the induction of the adaptive humoral response. We observed a peak in the transcription of antibody heavy chain genes in PBMC based on RNA-Seq, coinciding with a peak of the frequency of ASCs in PBMC as measured by CyTOF. This presumed peak in antibody production was accompanied by a significant increase of vaccine-specific antibody titers in plasma. As expected, titers peaked later (on day 14) due to the longer half-life of circulating antibodies resulting in a continued increase of antibody titers as long as ASCs are active. This demonstrates that circulating ASC and gene characteristics of early ASC responses precede and correlate with serological responses.

In addition to the similarities of the responses to Tdap boost in aP- versus wP-primed individuals, we also noted a number of differences. In terms of humoral responses, while there were no differences in overall IgG titers, there were differences in an IgG subtype and antigen-specific fashion: aP-primed individuals possessed higher IgG4 response against FHA and IgG3 response against FIM2/3. This difference was observed both at the peak of the response on day 14 and on the day prior to vaccination, suggesting an imprinted memory B cell response from prior vaccination. Given that the antibodies of the IgG4 isotype have limited or no opsonization function, it is plausible to hypothesize that its higher levels against the FHA antigen in aP-primed individuals, even before vaccination, could be linked with reduced ability to prevent asymptomatic infection or colonization (12). In future studies, it will be of interest to evaluate opsonization titers. Moreover, we found a negative correlation between high IgG4 titers for FHA prior to boosting and expression of a gene module 14 days after boost that is significantly induced in wP individuals and contains the chemokine CXCL5 and several genes associated with platelets. While it is tempting to speculate that the 2 are causally linked, additional studies will be needed to examine mechanistic links between them. Moreover, the fact that the trends for IgG4 responses were not observed uniformly for all pertussis vaccine antigens complicates the interpretation of these data.

Profiling of IgE responses revealed further differences between aP- and wP-primed individuals; while a subset of aP-primed individuals had substantial induced IgE response against several antigens, no such responses were observed for wP-primed individuals. This observation is in line with the previously reported polarization of aP-primed individuals toward Th2 responses (20), which are expected to correlate with increased IgE secretion; this was also observed in aP-primed infants (18). Observing differences in antibody isotype polarization decades after the initial priming is interesting and was also observed at the level of memory T cell responses (22). Although of high interest, the examination of memory T cell responses at the antigen-specific level is beyond the scope of this study and has already been addressed in our previous studies (22, 54).

In addition to differences in the antibody response, we also found disparities in the cellular response between aP- versus wP-primed individuals, which were driven by subsets of aP-primed individuals that had a higher inflammatory signature than other individuals, marked by increased expression of gene expression modules that included *ICAM1*, *NFKBIA*, and *CCL3*. The elevated expression of *CCL3* in a subset of aP-primed individuals on day 3 after boost was confirmed through multiple assays (RNA-Seq, PEA, ELISA), and these CCL3-high responders also included all individuals that showed boosted IgE responses to pertussis vaccine antigens.

Asymptomatic infection can occur even shortly after vaccination, and epidemiological data suggest up to a 6% chance for any person being infected during any given year (55). Thus, the cohort groups are unlikely to be cleanly distinguished, since they could have been exposed to the live bacteria — particularly the aP cohort, known to be associated with waning immunity and, therefore, decreased vaccine efficacy. Another possibility that could lead to differences among cohorts is the fact that different donors could have received different types of vaccines. However, we find this hypothesis unlikely, since dose or antigen composition were only marginally different among licensed DTaP at the time of priming and a recent benchmarking study found that available pertussis vaccines, despite changes in antigen composition, performed similarly among young children (56).

A caveat of this study is the fact that the aP vaccine was not licensed before 1996 and, therefore, that aP- and wP-primed cohorts are not matched in age. Hence, it is reasonable to speculate that the age of the subjects could contribute to the observable differences in the response to booster immunizations with Tdap. Previous studies (21, 22) showed that the type of vaccine given but not the age was associated with differential vaccine responses. Nevertheless, age and other demographics could be associated with the observed variability in responses and will need to be examined more closely in follow-up studies. Also, the lack of extensive clinical characterization of these cohorts is a caveat to this study.

Susceptibility to *Bordetella pertussis* infection differs widely, and host genetic variability could also contribute to the observed reemergence of whooping cough. In humans, there is limited knowledge about specific genetic variations that influence susceptibility to *Bordetella pertussis* infection. However, genetic susceptibilities in mice to pertussis infection have been extensively studied and implicated in the pathobiology of disease (57, 58). In future studies, it will be of interest to evaluate if genetic differences in humans can account for differential responses to booster vaccination.

Overall, our study is the first to our knowledge to provide a comprehensive picture of immune responses to Tdap booster vaccination for individuals primed with the aP or wP vaccine in childhood. The differences discovered between aP- versus wP-primed individuals will require further examination, and in our minds, they raise two hypotheses that could explain differences in vaccine efficacy: (a) aP-primed individuals show a higher level of IgG4 antibodies prior to and after boost as a result of initial priming, or (b) a subset of aP-primed individuals shows a differential response marked by high expression of proinflammatory genes (*IL6*, *IL1B*, *CCL3*, *CCL4*, *ICAM1*, and *NFKBIA*) after boost and shows higher levels of IgE against ACT, a *Bordetella pertussis* antigen not contained in the vaccine. While the number of individuals in this group is limited, and many genetic and environmental factors could lead to this differential response in a subgroup, we are specifically intrigued by the possibility that recent asymptomatic infection and colonization with *Bordetella pertussis* could be the cause for this differential response.

*Bordetella pertussis* colonization in humans was shown to induce a systemic immune response without causing clinical (whooping cough) symptoms in a controlled human infection study (59). Also, it is known that colonization can occur after aP but not wP vaccination in mice and baboon models (12, 30, 60), which would explain why the same subgroup is not seen in wP-primed individuals. This hypothesis will need to be addressed in future studies by directly assessing subclinical colonization using nasal swab collections for PCR-based detection of *Bordetella pertussis* and assessing its impact on the immune response.

The development of a new generation of pertussis vaccines is hindered by our lack of understanding of the molecular mechanisms of pertussis vaccination and the underlying immunologic basis of vaccine failure. The findings presented in this study give a better understanding of the immune signatures evoked by booster vaccination. Specifically, signatures from wP- but not aP-primed individuals, which are associated with improved protection, could provide insights for mechanistic readouts that can be utilized in the evaluation of new vaccination strategies. Additional studies with samples from different sets of individuals will be necessary to test and refine these hypotheses.

## Methods

### Study subjects.

We recruited 58 healthy adults from San Diego, California, USA. Demographic and all available clinical information can be found in Supplemental Table 6. Clinical data for each patient were collected by multiple approaches. Whenever possible, vaccination records were collected from study participants or parents/guardian as appropriate. For some donors, the original clinical vaccine record was not available or was incomplete, including the brand and composition of the vaccine, in which cases information was collected by the clinical coordinators through questionnaires, recording dates, and numbers of vaccination. All donors were recruited from the San Diego area and followed the recommended vaccination regimen (which is also necessary for enrollment in the California school system), which entails 5 DTaP or DTwP doses for children younger than 7 years old (3 doses at 2, 4, and 6 months and then 2 doses between 15 and 18 months and between 4 and6 years). As a primary vaccination, individuals of each group received exclusively DTaP or DTwP vaccines in infancy, and both groups received additional Tdap booster immunizations at 11–12 years and then potentially every 10 years, but no boost was administered at least in the previous 4 years prior to this study. Individuals who had been diagnosed with *Bordetella pertussis* infection at any given time in their life were excluded. Other exclusion criteria included: pregnancy at the start of the study (no record of previous pregnancy or vaccination administered during pregnancy was collected); presentation of severe disease or medical treatment that might interfere with study results; any vaccination in the last month and/or antibiotic use or fever (>100.4°F [38°C]). In all groups, male and female subjects were included equally and originally vaccinated with either DTwP or DTaP in infancy, received a booster vaccination with Tdap, and donated blood before the boost and 1, 3, 7, 14, 30, or 90 days after the boost. Plasma for the same time point samples was collected after blood processing. The pertussis (P) compounds in these vaccines (wP and aP) were coadministered with diphtheria toxoid (d) and tetanus toxoid (T). Also, the capital and lowercase letters denote higher or lower proportions of the overall components between vaccines.

### Booster vaccination.

Participants received a booster vaccine (Adacel) with tetanus toxoid (T), reduced diphtheria toxoid (d), and acellular pertussis vaccine adsorbed (aP; Tdap). Each dose of Adacel vaccine (0.5 mL) contains the following active ingredients: Detoxified PT, 2.5 μg; FHA, 5 μg; PRN, 3 μg; FIM2/3, 5 μg; tetanus toxoid (T), 5 limits of flocculation (Lf); and diphtheria toxoid (d), 2 Lf. Other ingredients include 1.5 mg aluminum phosphate (0.33 mg of aluminum) as the adjuvant besides residual formaldehyde, glutaraldehyde, and phenoxyethanol.

### PBMC and plasma isolation.

Plasma was obtained by centrifugation (400*g* for 15 minutes at 4°C) of whole blood samples and collection of the upper layer, prior to PBMC isolation by density gradient according to the manufacturer’s instructions (Ficoll-Paque Plus, Amersham Biosciences) as previously described (61). Plasma was then cryopreserved at –80°C, and cells were cryopreserved in liquid nitrogen suspended in FBS containing 10% (vol/vol) DMSO (Sigma-Aldrich). Alternatively, 6 × 10^6^ PBMC from each sample were transferred directly to Qiazol reagent (Qiagen), resuspended, and immediately aliquoted and stored at –80°C until RNA-Seq downstream processing.

### Multiplex luminex immunoassays.

Antigen-specific antibody responses were measured through a modified multiplex Luminex assay as previously reported (22, 45). Pertussis (PTM, PT, inactivated PT), tetanus, and diphtheria proteins, PRN, FHA, FIM2/3, adenylate cyclase toxin (ACT), lipooligosaccharide (LOS), tetanus toxoid (T), and diphtheria toxoid (d), were purchased from List Biological Laboratory (Campbell) and Sigma-Aldrich. Inactivated Rubeola antigen (Edmonston strain), used as an internal vaccine control, was purchased from Meridian Life Science Inc., and common allergens (cat allergen, Fel d1; birch allergen, Bet v1; rye grass allergen, Lol p1), used as IgE-allergen controls, were purchased from Indoor Biotechnologies. Irrelevant protein OVA and PD1, obtained from Invivogen, were used as internal negative controls and were coupled to distinct fluorescent-barcoded MagPlex microspheres (Luminex Corporation). To generate 1% PFA PT, native PT (List Biological Laboratories) was incubated with formaldehyde (Sigma-Aldrich) for a final concentration of 1% v/v for 1 hour at 4°C. The inactivated toxin was then dialyzed using Zebaä spin desalting columns (Thermo Fisher Scientific), and protein concentration was determined via a micro BCAä protein assay (Thermo Fisher Scientific). Plasma from each individual or WHO *Bordetella pertussis* human serum reference standard (National Institute for Biological Standards and Control [NIBSC], 06/140) were mixed with an equimolar amount of each conjugated microsphere. The microspheres were then washed with a PBS-tween 20 buffer (Sigma-Aldrich) to release nonspecific antibodies, and bound antibodies were detected via anti–human IgG phycoerythrin (PE; clone JDC-10), anti–human IgG1-PE (clone HP6001), anti–human IgG2-PE (clone HP6025), and anti–human IgG3-PE (clone HP6050, all from Southern Biotech); anti–human IgG4-PE (clone HP6025, Abcam); or human IgE-PE (clone BE5, Thermo Fisher Scientific) to measure isotype or IgG subclass antigen-specific antibodies. Samples were subsequently analyzed on a Luminex FLEXMAP 3D instrument (Luminex Corporation). PT-, PRN-, and FHA-specific IgG^+^ beads were calculated as IU/mL based on the WHO reference serum. Other antigen-specific IgG that had no reference standard or antigen-specific IgE^+^, IgG1^+^, IgG2^+^, IgG3^+^, and IgG4^+^ beads are reported as log_10_ of the median fluorescent intensity (MFI).

### PEA.

Plasma samples from 8 aP and 10 wP donors were sent to Analysis Lab at Olink Proteomics for analysis of a total of 276 proteins using 3 panels of PEA. Briefly, plasma was incubated with paired oligonucleotide labeled antibodies that target specific proteins. Once the antibody recognizes the antigen, the proximity of the oligonucleotide tails allows formation of the DNA amplicon, which enables amplification by PCR. Detection of protein-specific PCR products by real-time PCR allows collection of Ct values that are then transformed to normalized protein expression units (NPX), which allow comparison of protein expression between samples (62). The commercially available PEA panels applied were: Immuno-Oncology, Immune Response, and Metabolism, which allowed the identification of a total of 276 proteins and soluble factors in plasma. Two of the aP-primed donors were excluded from the study due to high variability in the technical internal control. A total of 23 analytes were excluded based on the number of samples that presented values below the limit of detection (LOD) to avoid introduction of results bias due to a low number of data points (Supplemental Table 7). Finally, 12 duplicate analytes due to panel overlap remaining after LOD exclusion were evaluated for correlation as an internal quality control (Supplemental Figure 14), and only 1 of the analyses performed in duplicate was included in the final analytes list. The final number of analytes included in the study was 241 for a cohort of 16 donors (6 aP-primed and 10 wP-primed individuals). Those 241 analytes were further filtered by exclusion of proteins with a coefficient of variation over 10% (CV > 0.1), which reduced the list of analytes included in the study to 209.

### ELISA.

Plasma samples from 20 aP and 19 wP donors were measured by ELISA. CCL3/MIP-1α DuoSet ELISA and Human LIGHT/TNFSF14 Quantikine ELISA Kit (both from R&D Systems) were conducted following manufacturer instructions.

### CyTOF cell analysis.

PBMC were thawed and directly stained with the viability marker Cisplatin, followed by a surface antibody cocktail incubated for 30 minutes. Subsequently, and after washes, cells were fixed in PBS (Thermo Fisher Scientific) with 2% PFA (Sigma-Aldrich) overnight at 4°C. The following day, cells were stained with an intracellular antibody cocktail after permeabilization using saponin-based Perm Buffer (eBioscience). Before sample acquisition and additional washes after staining, cellular DNA was labeled with Cell-ID Intercalator-Ir (Fluidigm). Samples were kept in the pellet form and resuspend in 1:10 of EQ Beads (Fluidigm) in 1 mL of MiliQ water and then acquired using a Helios mass cytometer (Fluidigm). Antibodies used in CyTOF are listed in Supplemental Table 8. Automated gating analysis of CyTOF data was conducted using DAFi (39). Source code of DAFi is publicly available at GitHub with configuration and usage information (https://github.com/JCVenterInstitute/DAFi-gating/commit/242b905e39aaf372643e333d32de7968f2eb7885; https://github.com/JCVenterInstitute/DAFi-gating/commit/93265ebb506df6b4eb2f50bb7e4613ee59f9ebe3). The DAFi configuration files used in the analysis can be found in https://github.com/JCVenterInstitute/DAFi-gating/commit/88bcdd51aa491bfb6456c94e50a7f36db9b4a301 for reproducing the results. Cell frequencies of all the 21 distinct cell populations were obtained by DAFi, with the exception of ASCs, which were independently analyzed and calculated by manual gating analysis using the combination of CD45^+^Live^+^CD14^–^CD3^–^CD19^+^CD20^–^CD38^+^ antibody markers.

### RNA-Seq.

RNA-Seq was performed as described previously (35, 36). Briefly, RNA was extracted using the Qiagen miRNeasy Mini kit (Qiagen) with on-column DNase treatment (Qiagen), and 500 ng of total RNA was used as input for library preparation using the Illumina TruSeq Stranded mRNA Library Prep Kit (Illumina) as previously described. The libraries were sequenced on the HiSeq3000 (Illumina).

### RNA-Seq bioinformatics data analysis.

Raw sequencing reads were aligned to the hg19 reference using TopHat (v 1.4.1, library-type fr-secondstrand-C) (63). Gencode v.19 (obtained from UCSC Genome Browser; https://genome.ucsc.edu/index.html) was used for analysis. The HTSeq library htseq-count with union mode was used to quantify reads for all annotated genes. Raw counts were TPM-normalized for subsequent analysis steps. In-depth transcriptomic data analysis methods are described in Supplemental Methods.

### Data availability.

The RNA-Seq data can be found in Gene Expression Omnibus (GEO) (accession code GSE152683; https://www.ncbi.nlm.nih.gov/geo/query/acc.cgi?acc=GSE152683). Antibody and proteomics raw data can be found in Supplemental Data 3 and 4, respectively.

### Statistics.

Statistical analyses are detailed for each specific technique in the specific Methods section or in the figure legends, where each specific comparison is presented. Statistical tests were performed using GraphPad Prism 8.4 (GraphPad Software, www.graphpad.com) and Python SciPy implementation of 2-sided Mann-Whitney *U* test (for unpaired comparisons) or Wilcoxon test (for paired comparisons). Details pertaining to significance are also noted in the respective legends, and *P* < 0.05 defined as statistical significant.

### Study approval.

This study was performed with approvals from the IRB at the La Jolla Institute for Immunology (protocol no. VD-101). All participants provided written informed consent for participation, and clinical medical history was collected and evaluated.

## Author contributions

B. Peters and AS conceived the study, and RDSA, FS, and NK conducted the experiments. B. Peters, AS, RDSA, MP, and FS wrote the manuscript. MP conducted the bioinformatic analysis and statistical analysis, with the collaboration of MB and JB. YQ, AM, and RHS developed DAFi-automated gating for CyTOF analysis, conducted the analysis, and provided feedback on the manuscript. YT helped with the antibody panel design and operation of the CyTOF mass cytometer. MC and B. Pulendran performed and coordinated the RNA-Seq experiments and provided feedback on the manuscript. CDP, LAP, and APG ran the multiplex assays to evaluate antibody isotype and antigen specificity and provided feedback on the manuscript. B. Peters oversaw all the statistical analysis.

## Supplementary Material

Supplemental data

Supplemental data 1

Supplemental data 2

Supplemental data 3

Supplemental data 4

## Version 1 03/09/2021

In-Press Preview

## Version 2 04/08/2021

Electronic publication
