# Supplementary material for: A system-view of Bordetella pertussis booster vaccine responses in adults primed with whole-cell versus acellular vaccine in infancy
Source: JCI Insight. 2021 Apr 8;6(7):e141023. doi: 10.1172/jci.insight.141023 (PMC8119213; doi:10.1172/jci.insight.141023)
Supplement: Supplemental data [file jciinsight-6-141023-s087.pdf]

Supplementary Materials

Supplementary Figures

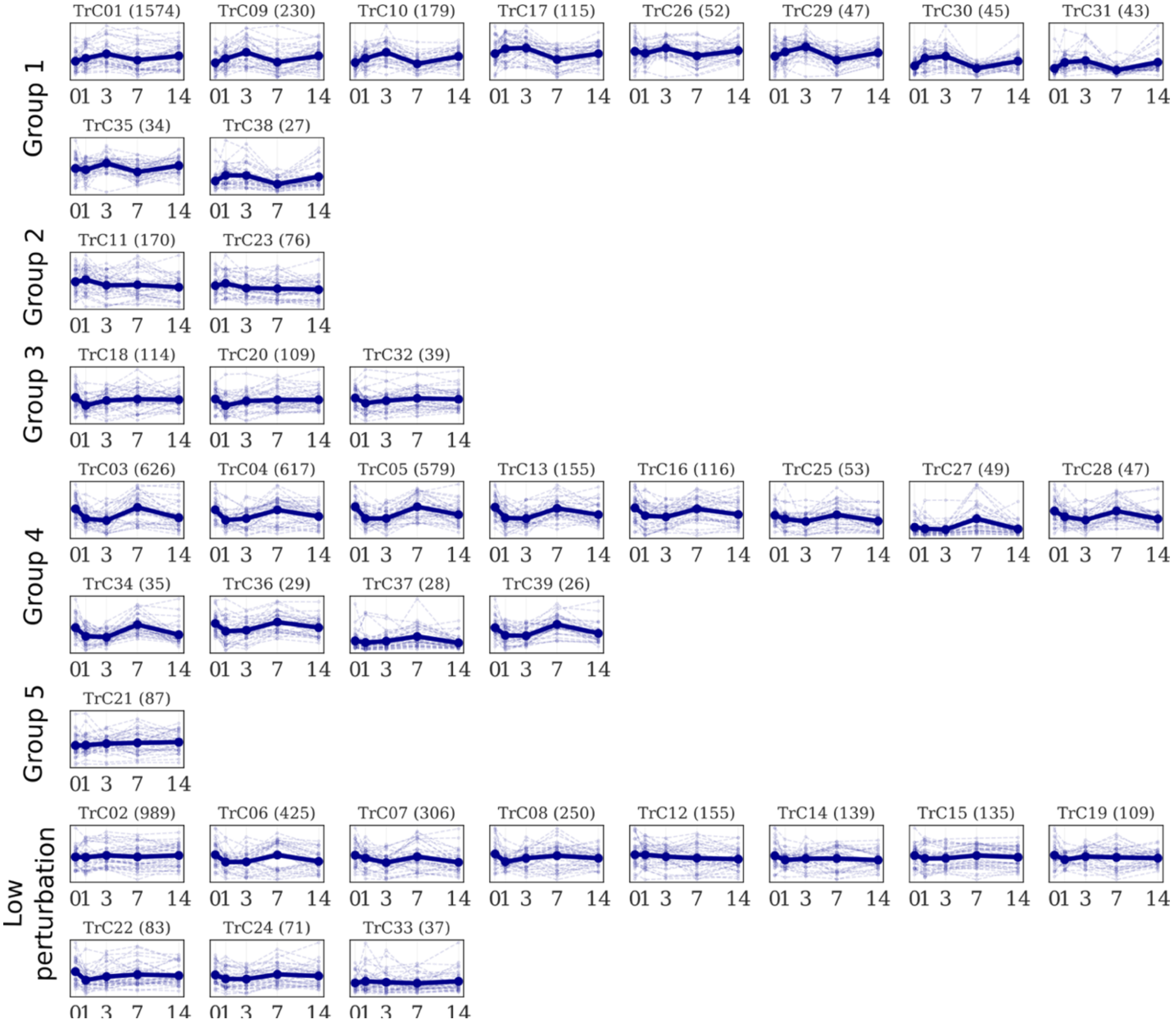

Supplementary Figure 1. Longitudinal patterns of transcriptomic clusters. Thick lines represent average of PC1 for a given cluster across all the profiled individuals. Thin lines represent PC1 values for a given individual. Clusters are grouped by longitudinal patterns. Data was generated with a total of 36 donors (n=36)

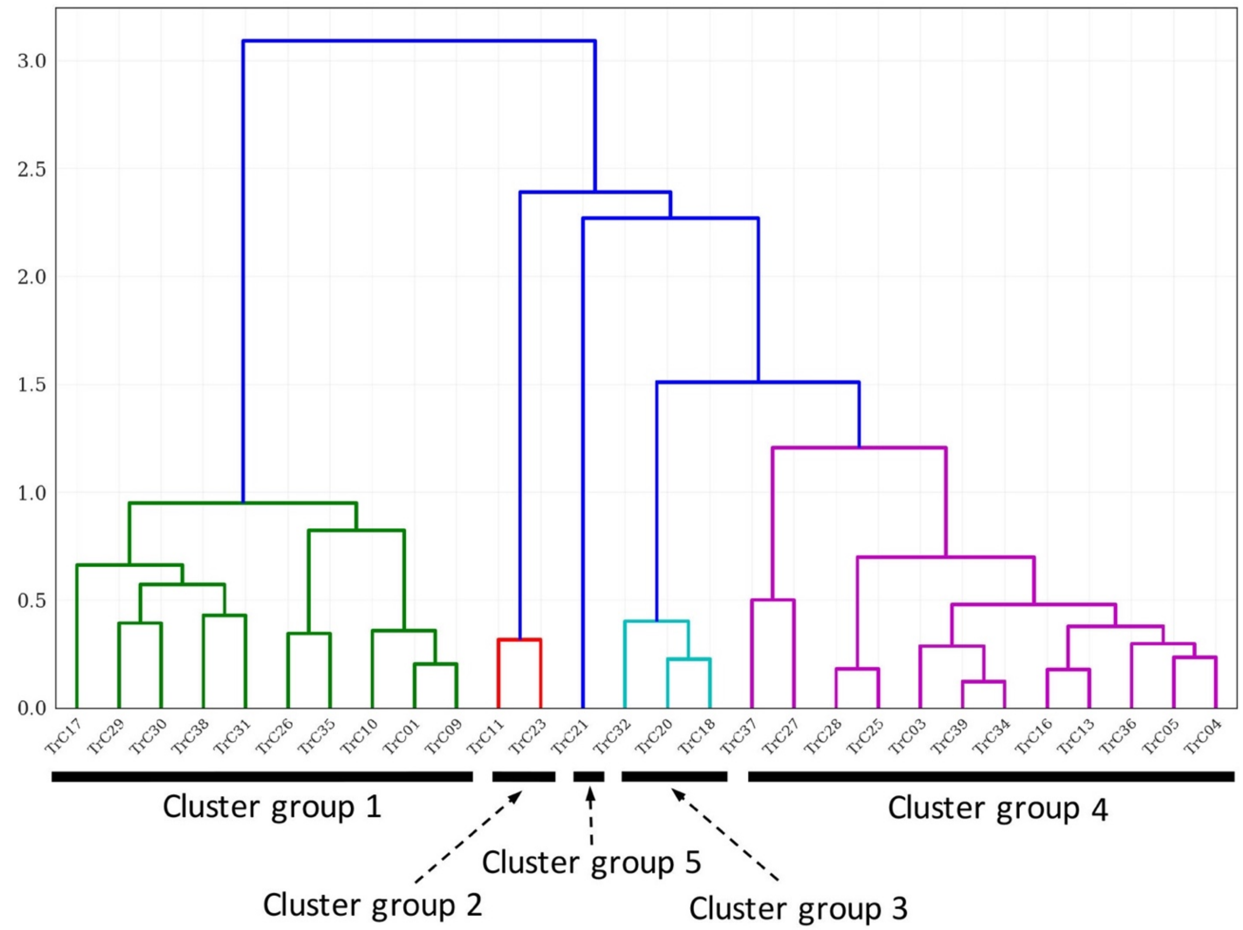

Supplementary Figure 2. Dendrogram for similarity of longitudinal patterns of transcriptomic clusters. Dendrogram was constructed using 'cityblock' distance metric and 'average' linkage method.

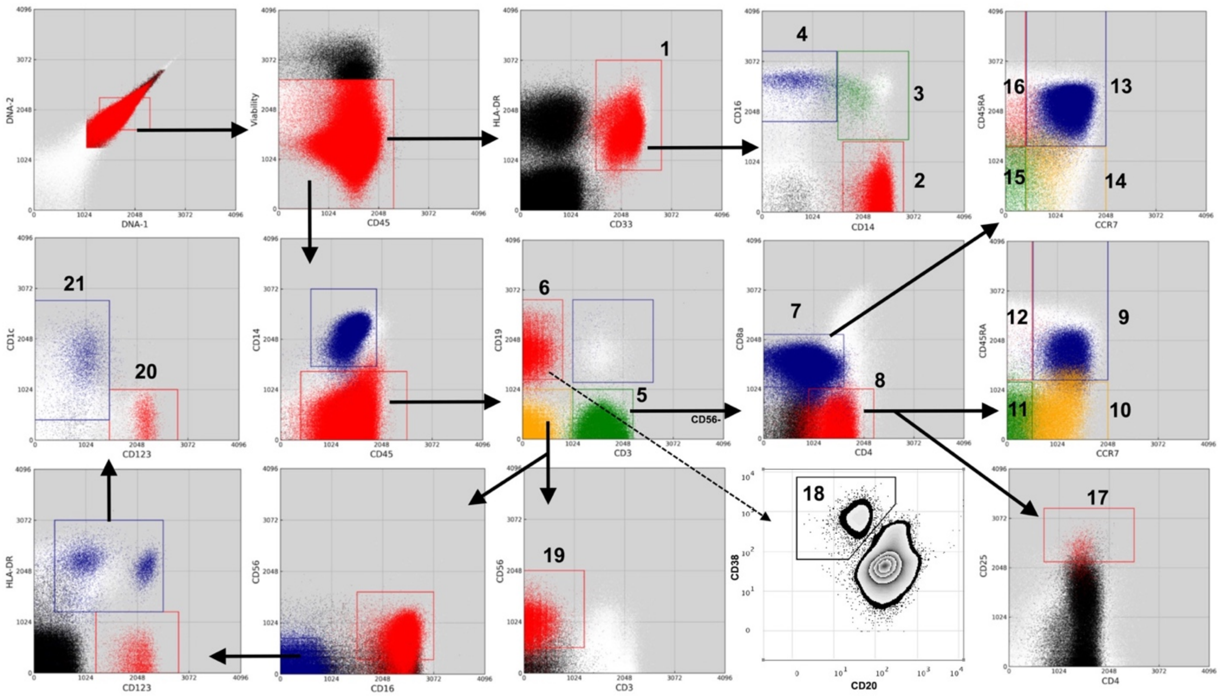

Supplementary Figure 3. Identification of immune cell subsets in human PBMCs by CyTOF. Each cell population was identified based on surface protein marker expression following the sequential dot-plot gating strategy by using manual gating analysis (cell population #18) or DAFi automated gating analysis (the other cell populations) . Each number (Gate ID) represents a major population or subset. 1) Monocytes, 2) Classical Monocytes, 3) Intermediate Monocytes, 4) Non-classical Monocytes, 5) CD3+ T cells, 6) B cells, 7) CD8+ T cells, 8) CD4+ T cells, 9) CD4+ naïve, 10) CD4+ Tcm, 11) CD4+ Tem, 12) CD4+ Temra, 13) CD8+ naïve, 14) CD8+ Tcm, 15) CD8+ Tem, 16) CD8+ Temra, 17) Tregs, 18) ASCs (Plasmablasts), 19) NK cells, 20) pDCs, 21) mDCs. Detailed gating strategy and analysis methods are described in Materials and Methods. Representative 2D plots are shown for illustration purpose.

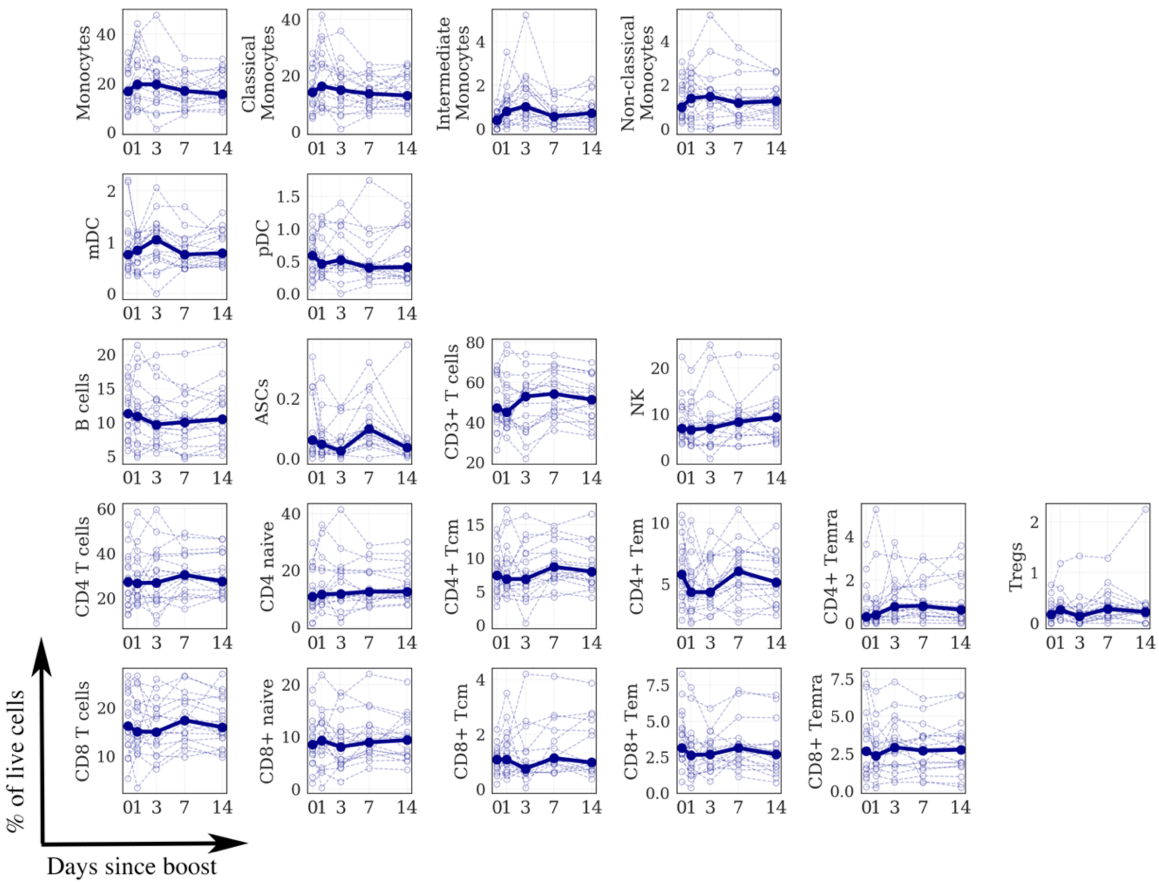

Supplementary Figure 4. Longitudinal patterns of cell populations frequency profiled with CyTOF. Each graphic represents the longitudinal kinetics of the percentage of live cells from total PBMC for each individual population (y axis) determined by high-dimensional automated gated analysis of data generated by CyTOF assay. Data are expressed as connected time points from day 0 to day 1, 3, 7 and 14 post boosts for each individual donor (thin lines) or the median of all (bold line). Data was generated with a total of 18 donors (n=18).

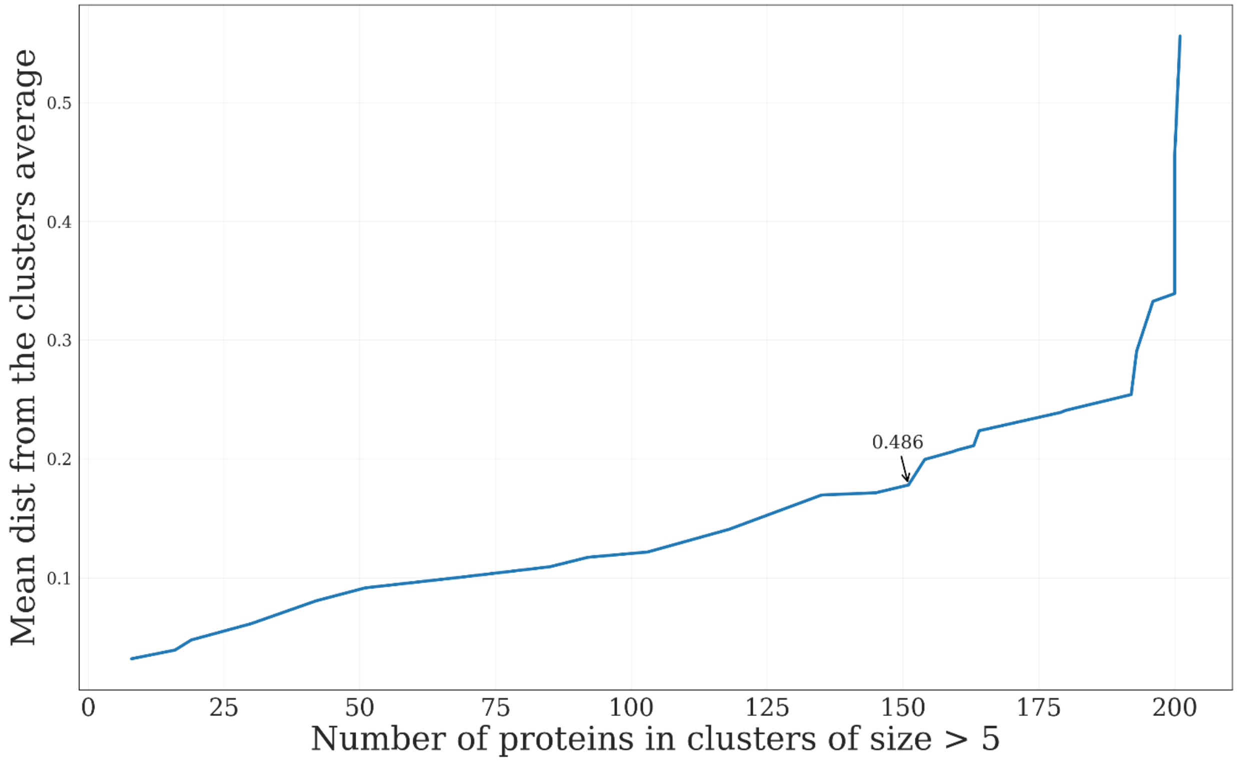

Supplementary Figure 5. Cut-off threshold for proteomics data clustering. X-axis indicates number of proteins in clusters of size greater than 5. Y-axis indicates measure of cluster tightness. More specifically, it is correlation of every protein in the cluster with the cluster's mean, averaging across all the proteins in clusters containing 5 or more genes. Annotated point indicates a threshold of 0.486 chosen for downstream analysis. It indicates a fraction of maximum inter-protein distance.

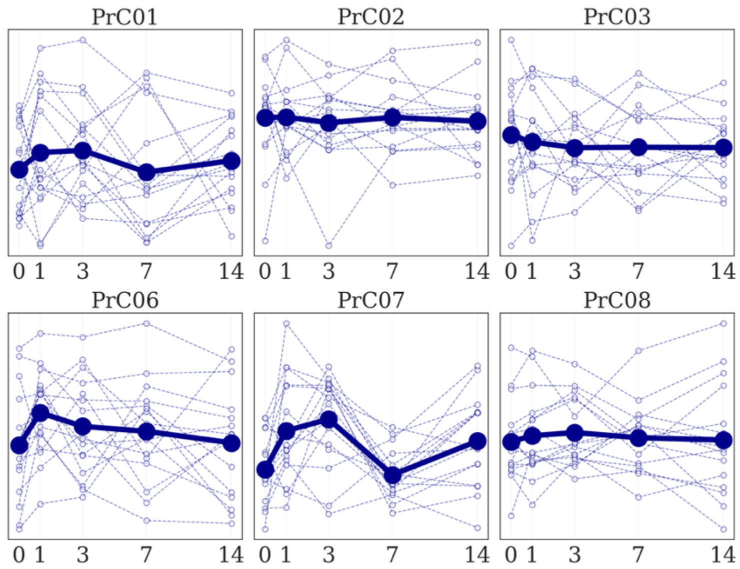

Supplementary Figure 6*. *Longitudinal patterns of serum protein clusters. Thick lines represent average of PC1 for a given cluster. Averaging is done across donors of the same cohort: either primed with aP or wP vaccine. Thin lines in the background represent PC1 for every individual. Number of proteins comprising a cluster is given in brackets in the title above every plot. Data are expressed as connected time points from day 0 to day 1, 3, 7 and 14 post boosts for each individual donor (thin lines) or the median of all (bold line). Data was generated with a total of 16 donors (n=16) after exclusion of 2 donors (See material and methods).

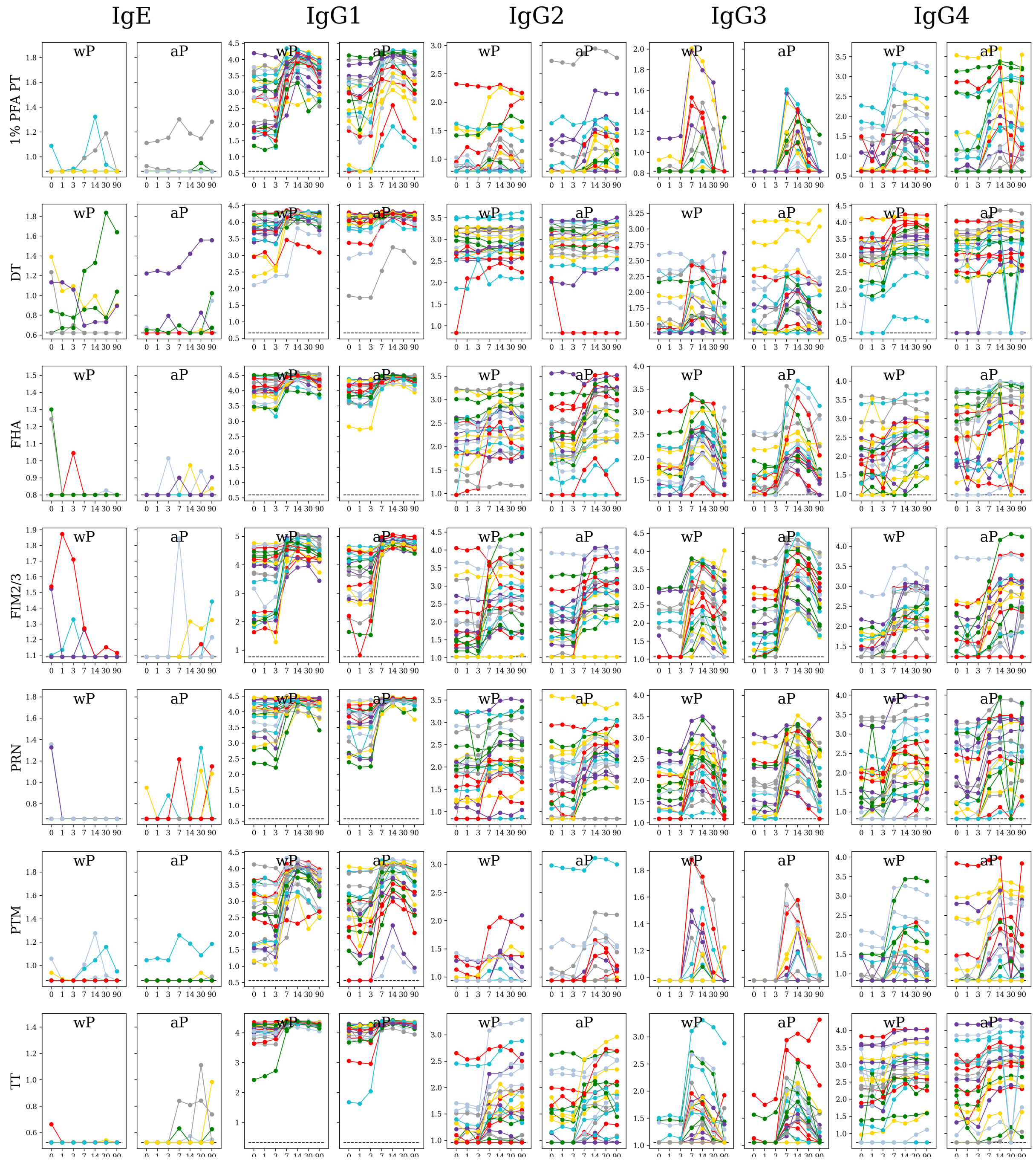

Supplementary Figure 7. Quantification of Tdap antigen-specific immunoglobulins. Profiles of IgE and IgG1-4 in seven antigens from Tdap vaccine: 1% PFA PT (pertussis toxoid denaturized in 1% paraformaldehyde), DT (diphtheria toxoid), FHA (Filamentous hemagglutinin), FIM2/3 (Fimbriae 2/3), PRN (pertactin), PTM (pertussis toxin mutant), TT (tetanus toxoid). Every immunoglobulin-antigen combination has two subplots, individually for DTwP- and DTaP-primed cohorts. Note an increase of IgG immunoglobulins against all the antigens at day 7. Y-axis indicates log10 of the IgE, IgG1, IgG2, IgG3, or IgG4 MFI. X-axis indicates days after vaccine boost (zero means pre-vaccination). Antigen-specific antibodies were evaluated for a total of 58 study participants (n=58).

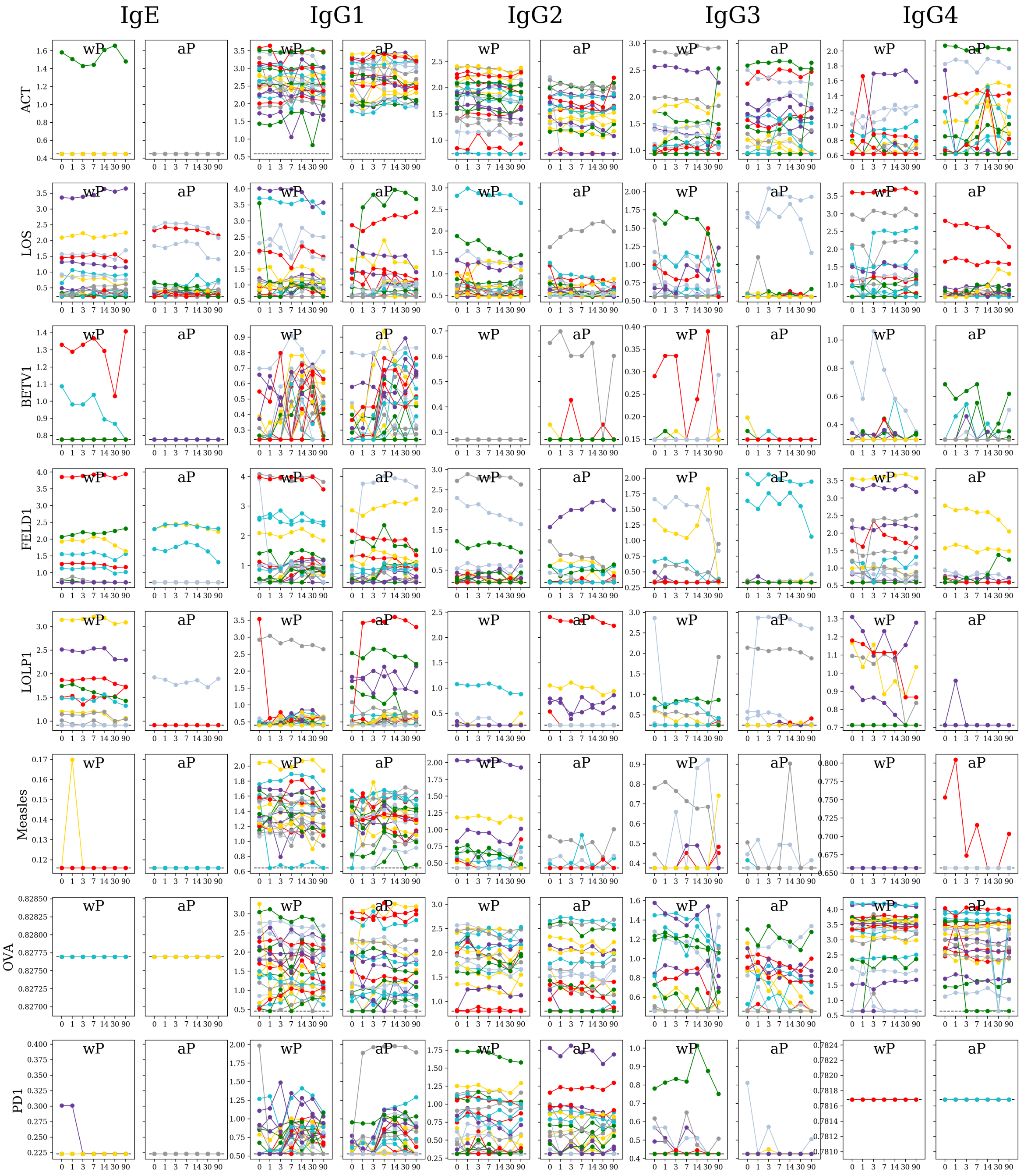

Supplementary Figure 8. Quantification of immunoglobulins against whole-cell vaccine antigens, allergens and control antigens. Profiles of IgE and IgG1-4 versus the following antigens are shown: ACT (Adenylate Cyclase, wP antigen), LOS (lipopolysaccharide, wP antigen), BETV1 (seasonal allergen), FelD1 (allergen), LOLP1 (seasonal allergen), Measles (control), OVA (control), PD1 (control). Every immunoglobulin-antigen combination has two subplots, individually for DTwP- and DTaP-primed cohorts. Data is clipped to limit of detection. Y-axis indicates log10 of the IgE, IgG1, IgG2, IgG3, or IgG4 MFI. X-axis indicates days after vaccine boost (zero means pre-vaccination). There is not consistent induction of immunoglobulin levels at day 7 against any of these antigens unlike in the previous figure. Antigen-specific antibodies were evaluated for a total of 58 study participants (n=58).

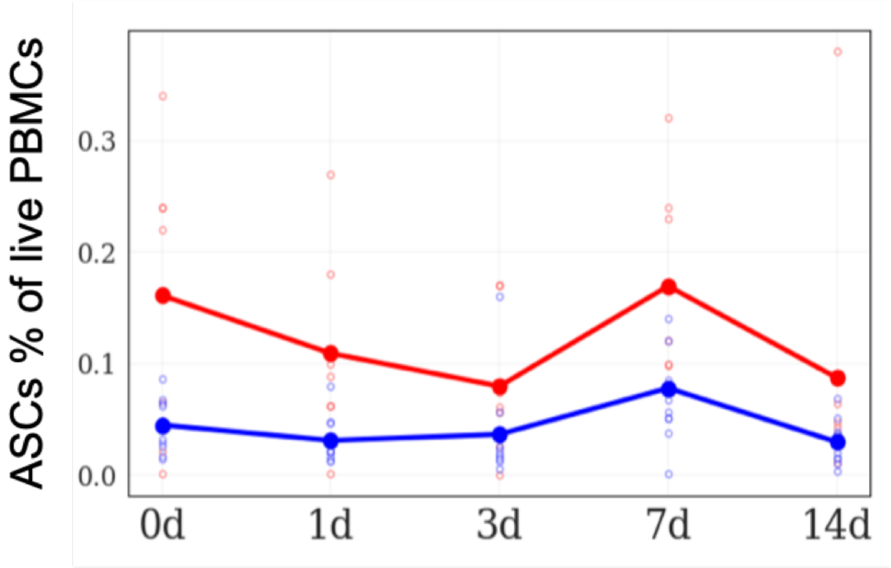

Supplementary Figure 9. Frequency of antibody secreting cells across timepoints in aP vs wP infancy-primed individuals. Frequency of antibody secreting cells (Y-axis) for each donor and timepoint (X-axis) is represented with an empty dot. Red dots belong to aP-primed individuals while blue dots represent wP-primed individuals in infancy. Bold lines and dots represent the median of the wP (Red) and aP (Blue). Data was generated with 10 wP and 8 aP donors for a total of 18 donors (n=18).

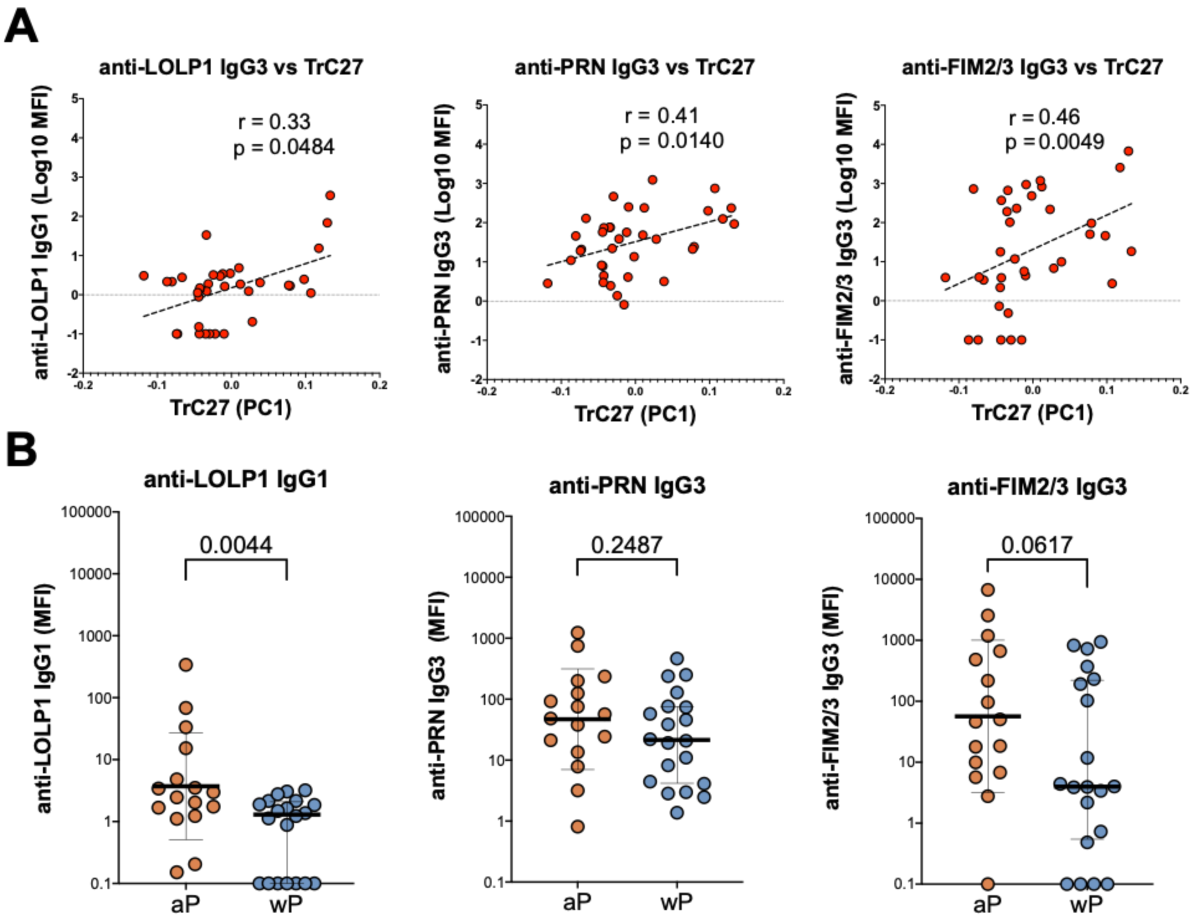

Supplementary Figure 10. Baseline correlations of antigen-specific antibodies and transcriptomic cluster 27 (TrC27). **A)** Graphs showing correlation of TrC27 (X-axis) and anti-LOLP1 IgG1, anti-PRN IgG3 and anti-FIM2/3 respectively (Y-axis). Units in the X axis consist in the individual PC1 for Cluster TrC27 while antibody concentration is expressed in the Y axis in mean fluorescence intensity (MFI). Statistical significance and r values were evaluated by Spearman correlation test with data from all the donors in the study for which antibody and transcriptomics data was obtained (n=36). **B)** Group comparison of baseline plasma levels of anti-LOLP1 IgG1, anti-PRN IgG3 and anti-FIM2/3. Individuals are represented with red dots (aP) or blue dots (wP). Statistical differences were evaluated using two-sided, non-parametric Mann-Whitney test. Data was generated with 20 wP and 16 aP donors for which transcriptomic and antibody data was available for a total of 36 donors (n=36). Geometric mean and SD are represented by error bars.

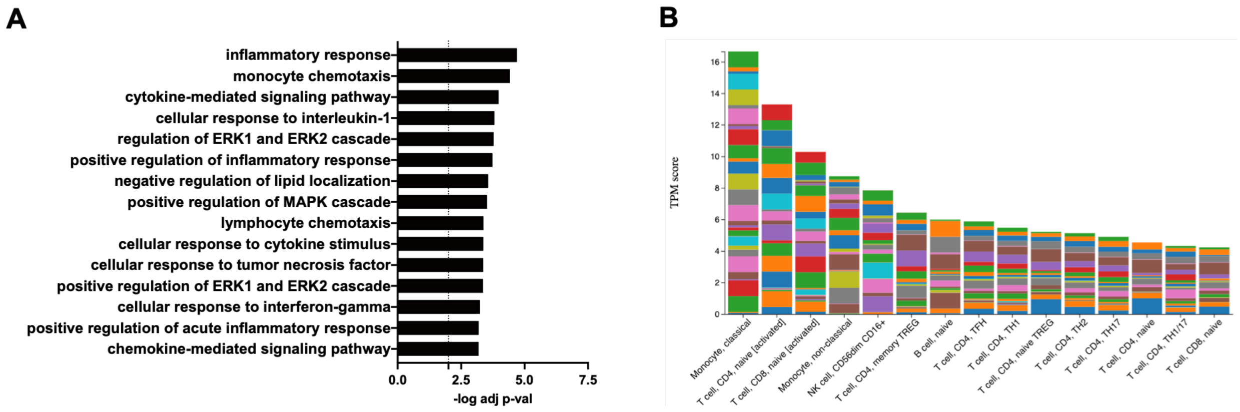

Supplementary Figure 11. Pathway analysis and cell source of the immune signature identified at day 3 in a subset of aP donors**. A)** Top 15 GO biological processes terms enriched in the transcriptomic signature (36 genes) identified in the subset of aP donors. Bars express -log adjusted p values for each term and dotted line represents an adjusted p value of 0.05. **B)** Cellular source of the transcriptional signatures identified using DICE database tool CellTypeScore (https://tools.dice-database.org/celltypescore/). X axis shows immune cell types while Y-axis presents TPM expression normalized per cell type. Different colors seen in the bars account for each individual transcript.

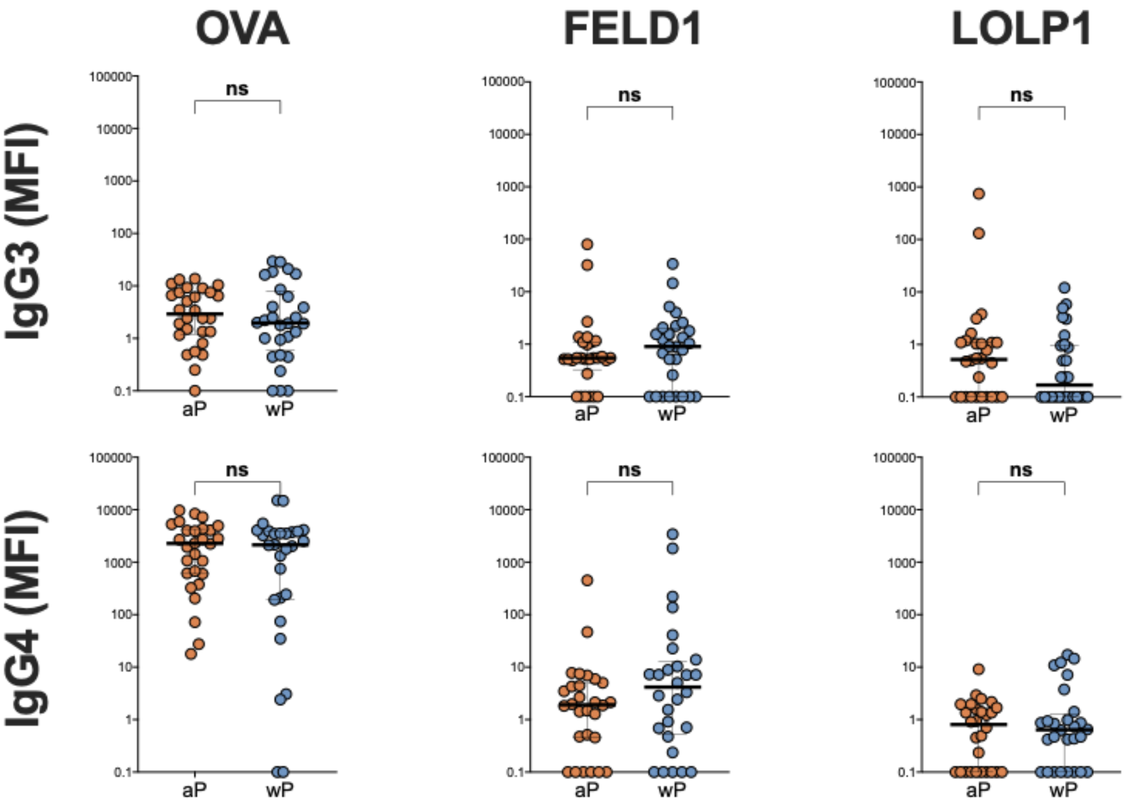

Supplementary Figure 12. Group comparison of baseline levels of non-vaccine antigen-specific IgG3 and IgG4. Baseline levels of antigen specific IgG3 and IgG4 against OVA, FELD1 and LOLP1. aP primed individuals are represented by red dots and wP primed individuals are represented in blue dots. Y-axis represents concentration of each antibody in mean fluorescence intensity a (MFI) in log scale. Data was generated for a total of 58 donors consisting in 28 for aP and 30 for wP primed individuals (n=58). Statistical differences were evaluated using a two-sided, non-parametric Mann-Whitney test. Bars and error bars represent geometric mean an SD.

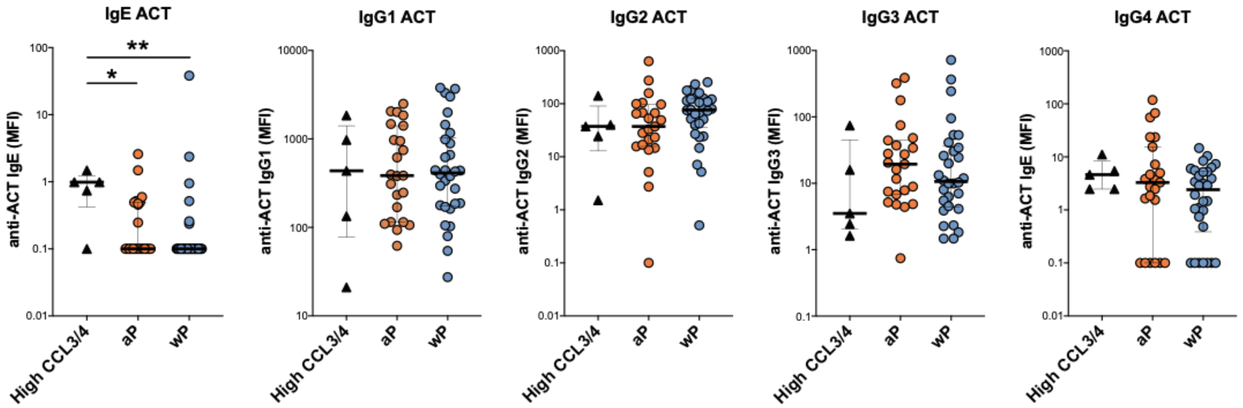

Supplementary Figure 13. Baseline levels of ACT-specific antibodies in aP vs wP-primed individuals and a subset of aP donors with high inflammatory profile at days 3 and 7 post boost. IgE, IgG1, IgG2, IgG3 and IgG4 levels of ACT-specific antibodies prior to Tdap booster vaccination. aP primed individuals are represented by red dots and wP primed individuals are represented in blue dots. aP donors with a differential cellular response at days 3- and 7- days post boost, are represented with black triangles. Y-axis represents concentration of each antibody in mean fluorescence intensity a (MFI) in log scale. Statistical differences were evaluated using a two-sided, non-parametric Mann-Whitney test (*p=0.0215, **p=0.0055). Data was generated with a total of 58 donors (n=58) divided in three groups of 5 High CCL3/CCL4 aP donors, 23 aP donors and 30 wP donors. Bars and error bars represent geometric mean an SD.

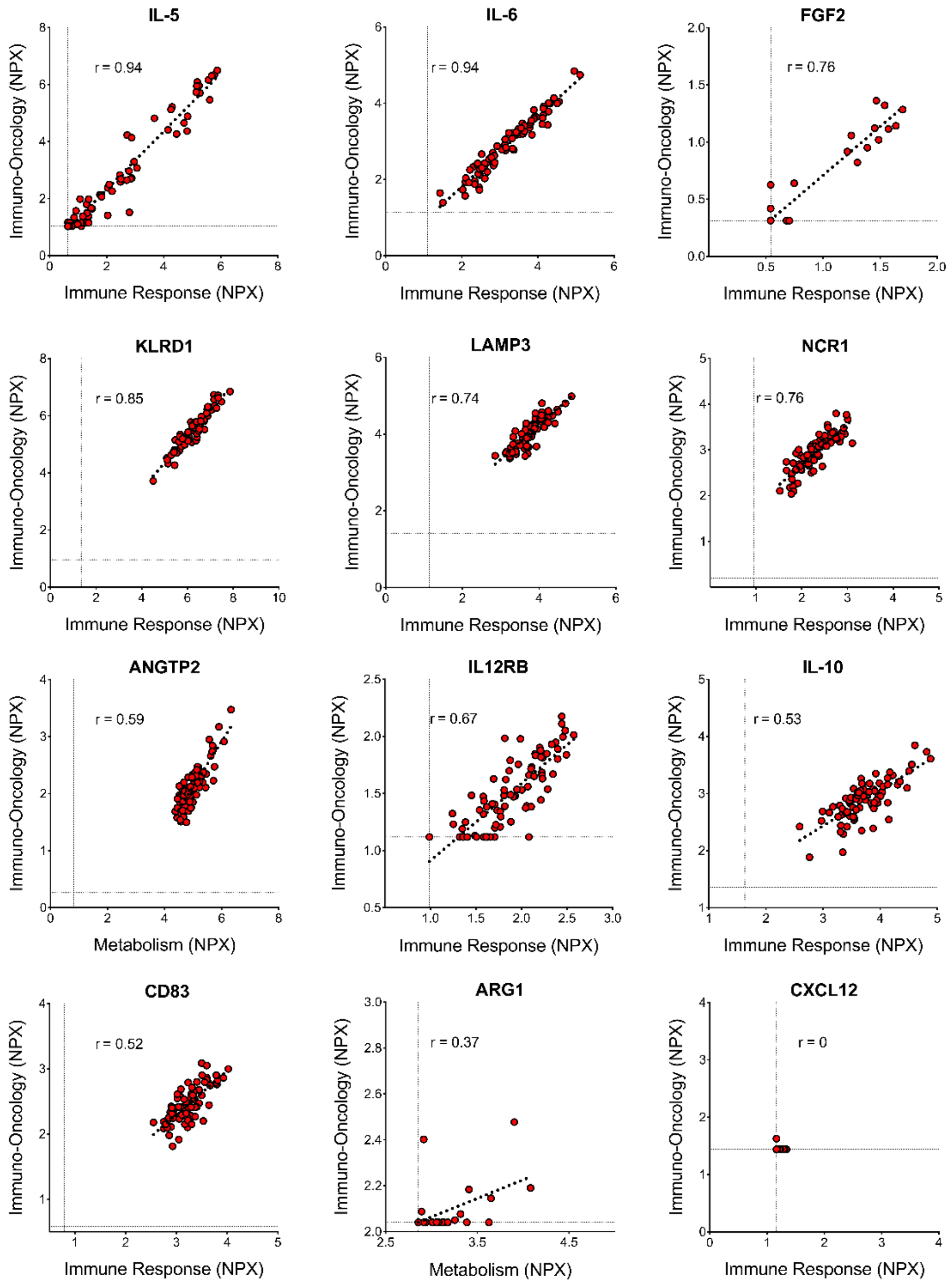

Supplementary Figure 14. Correlation of duplicate analytes included in the proteomics analysis. Each axis contains the normalized protein expression values (NPX) obtained by commercially available panels used to evaluate their presence in human plasma of 16 donors (n=16). Dotted line represents the limit of detection (LOD) of each analyte on each proximity extension assay panel (PEA). Correlations of the values obtained on each panel were performed with Spearman correlation test and r values for each individual analyte are represented on each graph. Values of Spearman r < 0.52 are obtained in analytes with the majority of NPX values equal or close to the LOD as observed in ARG1 and CXCL12. Data was generated with a total of 16 donors (n=16).

Supplementary Tables

Supplementary Table 1 – Breakdown of assays performed per donor

Supplementary Table S1	Assays performed							
COUNT	DONOR ID	GROUP	SERUM IMMUNOGLOBULINS	RNAseq	PLASMA PROTEOMICS	CyTOF	PLASMA ELISA	# ASSAYS
1	2730 *	aP	TRUE	TRUE	TRUE	TRUE	TRUE	5
2	2886 ◊	aP	TRUE	TRUE	TRUE	TRUE	TRUE	5
3	2902 *	aP	TRUE	TRUE	TRUE	TRUE	TRUE	5
4	2903 ⊙	aP	TRUE	TRUE	TRUE	TRUE	TRUE	5
5	2904 ◊	aP	TRUE	TRUE	TRUE	TRUE	TRUE	5
6	2912 *	aP	TRUE	TRUE	TRUE	TRUE	TRUE	5
7	2915 *	aP	TRUE	TRUE	TRUE	TRUE	TRUE	5
8	2928 *	aP	TRUE	TRUE	TRUE	TRUE	TRUE	5
9	1829 *	wP	TRUE	TRUE	TRUE	TRUE	TRUE	5
10	2383 *	wP	TRUE	TRUE	TRUE	TRUE	TRUE	5
11	2687 *	wP	TRUE	TRUE	TRUE	TRUE	TRUE	5
12	2691 *	wP	TRUE	TRUE	TRUE	TRUE	TRUE	5
13	2696 *	wP	TRUE	TRUE	TRUE	TRUE	TRUE	5
14	2702 *	wP	TRUE	TRUE	TRUE	TRUE	TRUE	5
15	2703 *	wP	TRUE	TRUE	TRUE	TRUE	TRUE	5
16	2726 *	wP	TRUE	TRUE	TRUE	TRUE	TRUE	5
17	2732 *	wP	TRUE	TRUE	TRUE	TRUE	TRUE	5
18	2738 *	wP	TRUE	TRUE	TRUE	TRUE	TRUE	5
19	2685 *	aP	TRUE	TRUE	FALSE	FALSE	TRUE	3
20	2698 *	aP	TRUE	TRUE	FALSE	FALSE	TRUE	3
21	2728 *	aP	TRUE	TRUE	FALSE	FALSE	TRUE	3
22	2736 *	aP	TRUE	TRUE	FALSE	FALSE	TRUE	3
23	2900 *	aP	TRUE	TRUE	FALSE	FALSE	TRUE	3
24	2901 *	aP	TRUE	TRUE	FALSE	FALSE	TRUE	3
25	2923	aP	TRUE	TRUE	FALSE	FALSE	TRUE	3
26	2935 *	aP	TRUE	TRUE	FALSE	FALSE	TRUE	3
27	1655 *	wP	TRUE	TRUE	FALSE	FALSE	TRUE	3
28	1832 *	wP	TRUE	TRUE	FALSE	FALSE	TRUE	3
29	2704 *	wP	TRUE	TRUE	FALSE	FALSE	TRUE	3
30	2706 *	wP	TRUE	TRUE	FALSE	FALSE	TRUE	3
31	2707 *	wP	TRUE	TRUE	FALSE	FALSE	TRUE	3
32	2719 *	wP	TRUE	TRUE	FALSE	FALSE	TRUE	3
33	2884 *	wP	TRUE	TRUE	FALSE	FALSE	TRUE	3
34	1687 *	wP	TRUE	TRUE	FALSE	FALSE	FALSE	2
35	2686 *	wP	TRUE	TRUE	FALSE	FALSE	FALSE	2
36	2699 *	wP	TRUE	TRUE	FALSE	FALSE	FALSE	2
37	2921 *	aP	TRUE	FALSE	FALSE	FALSE	TRUE	2
38	2968 *	aP	TRUE	FALSE	FALSE	FALSE	TRUE	2
39	2997 *	aP	TRUE	FALSE	FALSE	FALSE	TRUE	2
40	2998 *	aP	TRUE	FALSE	FALSE	FALSE	TRUE	2
41	3000 *	aP	TRUE	FALSE	FALSE	FALSE	TRUE	2
42	2689 *	aP	TRUE	FALSE	FALSE	FALSE	FALSE	1
43	2887 *	aP	TRUE	FALSE	FALSE	FALSE	FALSE	1
44	2890 *	aP	TRUE	FALSE	FALSE	FALSE	FALSE	1
45	2922 *	aP	TRUE	FALSE	FALSE	FALSE	FALSE	1
46	2952 *	aP	TRUE	FALSE	FALSE	FALSE	FALSE	1
47	2976 *	aP	TRUE	FALSE	FALSE	FALSE	FALSE	1
48	2995 *	aP	TRUE	FALSE	FALSE	FALSE	FALSE	1
49	2500 *	wP	TRUE	FALSE	FALSE	FALSE	FALSE	1
50	2688 ◊	wP	TRUE	FALSE	FALSE	FALSE	FALSE	1
51	2690	wP	TRUE	FALSE	FALSE	FALSE	FALSE	1
52	2693 *	wP	TRUE	FALSE	FALSE	FALSE	FALSE	1
53	2729 *	wP	TRUE	FALSE	FALSE	FALSE	FALSE	1
54	2731 *	wP	TRUE	FALSE	FALSE	FALSE	FALSE	1
55	2883 *	wP	TRUE	FALSE	FALSE	FALSE	FALSE	1
56	2893 *	wP	TRUE	FALSE	FALSE	FALSE	FALSE	1
57	2895 *	wP	TRUE	FALSE	FALSE	FALSE	FALSE	1
58	2899 *	wP	TRUE	FALSE	FALSE	FALSE	FALSE	1
			58	36	18	18	38	58

* antibody evaluation in plasma at 0, 1, 3, 7, 14, 30- and 90-120-days post boost

◊ antibody evaluation in plasma at 0, 1, 3, 7, 14- and 30-days post boost

⊙antibody evaluation in plasma at 0, 1, 3, 7, 14- and 90-120-days post boost

Supplementary Table 2 – Transcriptomic clusters perturbation Wilcoxon p-values

	Wilcoxon p-value		
	all	wP	aP
**TrC01**	6.72E-04	2.27E-02	3.36E-03
**TrC02**	1.10E-01	3.30E-01	7.71E-02
**TrC03**	5.52E-05	1.12E-02	3.36E-03
**TrC04**	1.03E-03	3.82E-02	1.13E-02
**TrC05**	1.53E-04	7.78E-03	1.13E-02
**TrC06**	1.09E-02	5.83E-02	4.86E-02
**TrC07**	1.81E-02	1.79E-02	1.27E-01
**TrC08**	2.91E-02	1.62E-01	8.73E-02
**TrC09**	1.42E-04	1.34E-02	3.36E-03
**TrC10**	8.69E-06	2.54E-03	3.36E-03
**TrC11**	6.65E-04	1.52E-02	3.10E-02
**TrC12**	2.32E-01	7.96E-02	6.05E-01
**TrC13**	8.20E-05	5.73E-03	7.54E-03
**TrC14**	3.89E-01	3.91E-01	1.98E-01
**TrC15**	1.09E-02	9.10E-02	8.05E-02
**TrC16**	7.20E-05	4.90E-03	1.09E-02
**TrC17**	5.48E-06	1.09E-03	5.30E-02
**TrC18**	6.69E-03	4.70E-03	1.37E-01
**TrC19**	3.65E-01	2.68E-01	3.45E-01
**TrC20**	5.42E-03	4.41E-03	5.29E-01
**TrC21**	4.37E-02	4.41E-03	8.05E-02
**TrC22**	7.55E-03	1.74E-02	4.55E-01
**TrC23**	8.42E-03	5.42E-03	5.49E-01
**TrC24**	5.10E-03	2.43E-02	4.46E-02
**TrC25**	1.15E-04	1.62E-02	5.25E-03
**TrC26**	1.88E-04	1.79E-02	6.85E-03
**TrC27**	5.48E-06	1.09E-03	3.36E-03
**TrC28**	1.42E-04	1.91E-02	3.36E-03
**TrC29**	5.48E-06	6.47E-03	3.36E-03
**TrC30**	3.04E-05	1.09E-03	3.36E-03
**TrC31**	2.32E-05	1.09E-03	8.33E-03
**TrC32**	7.55E-03	9.34E-03	4.74E-01
**TrC33**	4.99E-01	3.60E-01	2.32E-01
**TrC34**	2.95E-05	4.53E-03	6.24E-03
**TrC35**	3.67E-05	6.08E-03	6.24E-03
**TrC36**	9.36E-05	5.14E-03	7.71E-02
**TrC37**	1.39E-03	1.52E-02	9.46E-02
**TrC38**	1.47E-05	1.09E-03	7.42E-02
**TrC39**	8.69E-06	3.31E-03	3.36E-03

Supplementary Table 3. Identification of immune cell subsets in human PBMCs by CyTOF

Gate ID	Population name	Phenotype	Gating Method
N/A	Intercalator staining	DNA-1 (191Ir) vs DNA -2 (193Ir)⤵	Automated
N/A	Hematopoietic live cells staining	CD45+ (89Y) vs Viable- (195Pt)⤵	Automated
1	Monocytes	↳HLA-DR+CD33+	Automated
2	Classical Monocytes	↳HLA-DR+CD33+CD14+CD16-	Automated
3	Intermediate Monocytes	↳HLA-DR+CD33+CD14+CD16+	Automated
4	Non-classical Monocytes	↳HLA-DR+CD33+CD14-CD16+	Automated
5	CD3+ T cells	↳CD14-CD3+CD19-CD56-	Automated
6	B cells	↳CD14-CD3-CD19+	Automated
7	CD8+ T cells	↳CD14-CD3+CD19-CD56-CD4-CD8+	Automated
8	CD4+ T cells	↳CD14-CD3+CD19-CD56-CD4+CD8-	Automated
9	CD4+ naive	↳CD14-CD3+CD19-CD56-CD8-CD4+CD45RA+CCR7+	Automated
10	CD4+ Tcm	↳CD14-CD3+CD19-CD56-CD8-CD4+CD45RA-CCR7+	Automated
11	CD4+ Tem	↳CD14-CD3+CD19-CD56-CD8-CD4+CD45RA-CCR7-	Automated
12	CD4+ Temra	↳CD14-CD3+CD19-CD56-CD8-CD4+CD45RA+CCR7-	Automated
13	CD8+ naive	↳CD14-CD3+CD19-CD56-CD8+CD4-CD45RA+CCR7+	Automated
14	CD8+ Tcm	↳CD14-CD3+CD19-CD56-CD8+CD4-CD45RA-CCR7+	Automated
15	CD8+ Tem	↳CD14-CD3+CD19-CD56-CD8+CD4-CD45RA-CCR7-	Automated
16	CD8+ Temra	↳CD14-CD3+CD19-CD56-CD8+CD4-CD45RA+CCR7-	Automated
17	Tregs	↳CD14-CD3+CD19-CD56-CD4+CD8-CD25+	Automated
18	ASCs (Plasmablasts)	↳CD14-CD3-CD19+CD20-CD38+	Manual
19	NK	↳CD14-CD3-CD19-CD56+	Automated
20	pDCs	↳CD14-CD3-CD19-CD56-CD16-HLA-DR+CD1c-CD123+	Automated
21	mDCs	↳CD14-CD3-CD19-CD56-CD16-HLA-DR+CD1c+CD123-	Automated

Supplementary Table 4 – Cell frequencies vs transcriptomic cluster Spearman correlation r values

Cluster ID	Monocytes	CD33+HLADR+	Classical_Monocytes	Non-Classical_Monocytes	Intermediate_Monocytes	Bcells	CD3CD19	CD3CD19neg	CD3 Tcells	CD4Tcells	CD8Tcells	Tregs	TemraCD4	NaiveCD4	TemCD4	TcmCD4	TemraCD8	NaiveCD8	TemCD8	TcmCD8	NK	Basophils	mDC	pDC
**TrC01**	-0.14	-0.14	-0.17	-0.01	0.12	-0.21	-0.02	0.09	0.09	0.11	-0.02	-0.08	0.19	0.06	0.09	0.17	0.14	-0.09	0.09	0.16	-0.15	0.07	0.10	0.07
**TrC02**	-0.12	-0.12	-0.16	-0.08	0.19	-0.32	0.06	0.16	-0.02	-0.13	0.02	-0.07	0.04	-0.21	-0.03	0.13	0.12	-0.03	0.14	0.30	-0.11	0.01	0.12	-0.04
**TrC03**	0.13	0.13	0.16	0.00	-0.09	0.21	0.05	-0.11	-0.07	-0.07	0.07	0.13	-0.19	-0.09	-0.03	-0.06	-0.09	0.09	-0.03	-0.03	0.15	-0.06	-0.13	-0.12
**TrC04**	-0.10	-0.10	-0.07	-0.07	-0.28	0.27	0.07	-0.08	0.07	0.10	0.07	0.00	-0.06	0.19	-0.01	-0.13	-0.18	0.16	-0.15	-0.25	0.18	-0.11	-0.24	-0.05
**TrC05**	-0.20	-0.20	-0.17	-0.20	-0.22	0.13	0.18	-0.08	0.10	0.09	0.24	0.01	-0.17	0.10	-0.03	0.04	-0.19	0.30	-0.07	0.06	0.15	-0.11	-0.28	-0.16
**TrC06**	0.28	0.28	0.32	0.17	-0.07	0.27	-0.08	-0.12	-0.09	-0.07	-0.09	0.13	-0.11	-0.01	-0.04	-0.20	-0.07	-0.06	-0.08	-0.30	0.14	-0.01	0.01	0.03
**TrC07**	0.54	0.54	0.56	0.20	0.16	0.20	-0.11	-0.15	-0.30	-0.28	-0.12	0.18	-0.30	-0.32	-0.08	-0.16	-0.01	-0.13	-0.02	-0.09	0.04	0.07	0.15	0.03
**TrC08**	0.05	0.05	0.08	0.09	-0.20	0.27	-0.04	-0.10	0.04	0.08	-0.06	0.02	0.03	0.19	-0.01	-0.20	-0.08	0.00	-0.13	-0.37	0.13	-0.08	-0.08	0.09
**TrC09**	-0.09	-0.10	-0.13	0.05	0.08	-0.14	-0.06	0.08	0.09	0.14	-0.08	-0.08	0.26	0.14	0.10	0.09	0.15	-0.14	0.07	-0.01	-0.15	0.04	0.10	0.10
**TrC10**	-0.02	-0.03	-0.05	0.10	0.07	-0.07	-0.09	0.07	0.03	0.11	-0.17	-0.10	0.26	0.15	0.07	-0.01	0.11	-0.19	-0.01	-0.13	-0.14	0.06	0.13	0.14
**TrC11**	0.74	0.74	0.76	0.30	0.28	0.20	-0.17	-0.12	-0.43	-0.40	-0.28	0.15	-0.31	-0.46	-0.09	-0.22	0.05	-0.29	-0.06	-0.13	0.03	0.12	0.33	0.14
**TrC12**	0.69	0.69	0.72	0.35	0.20	0.24	-0.20	-0.13	-0.34	-0.30	-0.31	0.16	-0.20	-0.30	-0.09	-0.27	0.05	-0.31	-0.10	-0.30	0.06	0.10	0.31	0.19
**TrC13**	-0.19	-0.18	-0.18	-0.22	-0.05	-0.04	0.25	-0.01	0.02	-0.10	0.26	0.00	-0.25	-0.13	-0.13	0.07	-0.12	0.29	0.01	0.30	0.09	-0.09	-0.20	-0.20
**TrC14**	0.57	0.56	0.59	0.31	0.11	0.20	-0.17	-0.12	-0.26	-0.24	-0.27	0.17	-0.14	-0.21	-0.06	-0.26	0.03	-0.26	-0.09	-0.34	0.08	0.03	0.26	0.17
**TrC15**	-0.13	-0.12	-0.15	-0.11	0.17	-0.33	0.12	0.14	-0.04	-0.18	0.06	0.01	-0.01	-0.28	-0.06	0.12	0.09	0.00	0.13	0.36	-0.08	-0.01	0.03	-0.13
**TrC16**	-0.03	-0.02	-0.04	-0.09	0.16	-0.22	0.15	0.10	-0.11	-0.28	0.10	0.05	-0.18	-0.39	-0.12	0.07	0.02	0.07	0.12	0.36	0.00	-0.06	0.00	-0.16
**TrC17**	0.70	0.71	0.72	0.44	0.30	0.21	-0.24	-0.04	-0.40	-0.33	-0.40	0.04	-0.10	-0.34	-0.07	-0.29	0.13	-0.41	-0.08	-0.35	-0.02	0.10	0.39	0.26
**TrC18**	-0.32	-0.34	-0.31	-0.12	-0.30	0.20	0.09	-0.05	0.23	0.30	0.09	-0.11	0.12	0.46	0.01	-0.08	-0.19	0.19	-0.16	-0.25	0.11	-0.10	-0.26	0.02
**TrC19**	0.20	0.20	0.22	0.22	-0.09	0.29	-0.08	-0.09	-0.04	0.02	-0.16	0.04	0.06	0.12	-0.01	-0.22	-0.01	-0.13	-0.12	-0.41	0.09	-0.06	0.02	0.12
**TrC20**	-0.54	-0.56	-0.53	-0.35	-0.39	-0.02	0.06	-0.14	0.43	0.58	0.24	-0.06	0.14	0.66	0.17	0.24	-0.19	0.31	-0.11	0.05	-0.09	0.02	-0.27	0.02
**TrC21**	0.05	0.05	0.04	-0.06	0.04	-0.29	-0.14	0.10	-0.11	0.00	-0.12	0.01	0.09	-0.06	0.17	0.02	-0.25	-0.08	0.01	0.02	-0.10	0.22	0.13	0.13
**TrC22**	-0.19	-0.15	-0.22	0.23	0.04	0.07	0.17	0.70	-0.28	-0.36	-0.14	-0.41	0.09	-0.28	-0.03	-0.43	0.11	-0.16	0.12	-0.36	0.63	-0.47	-0.13	-0.20
**TrC23**	0.67	0.67	0.68	0.25	0.40	0.00	-0.13	-0.02	-0.49	-0.50	-0.28	0.12	-0.30	-0.62	-0.11	-0.15	0.07	-0.29	-0.03	0.08	-0.04	0.12	0.34	0.08
**TrC24**	-0.34	-0.32	-0.33	0.02	-0.08	0.74	0.36	0.16	-0.13	-0.04	0.05	-0.28	0.00	0.09	-0.22	-0.18	-0.03	0.15	-0.29	-0.04	0.24	-0.31	-0.45	-0.31
**TrC25**	0.32	0.33	0.36	0.20	-0.08	0.27	-0.16	-0.11	-0.13	-0.06	-0.12	0.19	-0.10	-0.03	0.07	-0.16	-0.02	-0.13	-0.02	-0.26	0.13	0.01	0.02	0.02
**TrC26**	0.07	0.07	0.08	0.18	-0.04	0.22	-0.06	-0.04	0.00	0.07	-0.17	-0.09	0.13	0.21	-0.01	-0.21	0.04	-0.14	-0.10	-0.35	-0.01	-0.06	0.04	0.11
**TrC27**	-0.09	-0.11	-0.06	-0.06	-0.24	0.30	0.05	-0.18	0.20	0.21	0.16	0.09	0.09	0.27	-0.03	0.03	-0.19	0.27	-0.01	-0.10	0.07	0.02	-0.31	-0.19
**TrC28**	0.37	0.38	0.38	0.12	0.21	-0.01	0.00	-0.03	-0.30	-0.37	-0.07	0.17	-0.25	-0.50	-0.07	-0.03	0.06	-0.13	0.07	0.19	0.04	-0.02	0.11	-0.09
**TrC29**	0.36	0.36	0.36	0.34	0.17	0.15	-0.16	0.01	-0.20	-0.08	-0.36	-0.04	0.15	-0.04	0.01	-0.20	0.11	-0.35	-0.09	-0.37	-0.05	0.02	0.22	0.20
**TrC30**	0.34	0.34	0.34	0.25	0.18	0.08	-0.19	-0.08	-0.12	0.02	-0.26	0.01	0.19	-0.03	0.10	0.00	0.17	-0.31	0.01	-0.14	-0.22	0.11	0.19	0.11
**TrC31**	0.20	0.18	0.20	0.02	0.12	0.22	-0.07	-0.08	-0.12	-0.03	-0.14	-0.18	-0.08	0.03	-0.15	-0.17	0.09	-0.10	-0.24	-0.25	-0.10	0.10	0.09	0.32
**TrC32**	-0.63	-0.66	-0.63	-0.52	-0.38	-0.30	0.04	-0.14	0.50	0.61	0.38	-0.01	0.08	0.61	0.24	0.44	-0.23	0.45	0.00	0.33	-0.16	0.09	-0.29	-0.01
**TrC33**	-0.09	-0.10	-0.05	-0.18	-0.08	0.15	0.07	-0.14	0.04	0.20	0.13	-0.06	0.08	0.14	0.20	0.11	0.10	0.11	0.14	0.12	-0.21	0.04	-0.26	-0.25
**TrC34**	-0.21	-0.20	-0.18	-0.17	-0.20	-0.02	0.08	0.04	0.06	0.07	0.14	0.14	0.03	0.00	0.05	0.12	-0.13	0.16	0.00	0.14	0.17	-0.12	-0.25	-0.22
**TrC35**	-0.13	-0.13	-0.13	0.06	-0.08	0.03	-0.04	0.03	0.12	0.15	-0.10	-0.14	0.22	0.30	0.01	-0.15	-0.06	-0.04	-0.08	-0.32	-0.04	0.04	0.01	0.16
**TrC36**	-0.64	-0.65	-0.62	-0.54	-0.36	-0.12	0.19	-0.10	0.39	0.46	0.39	0.02	0.02	0.52	0.09	0.30	-0.27	0.51	-0.14	0.32	-0.03	0.03	-0.37	-0.13
**TrC37**	0.11	0.11	0.12	0.17	-0.03	0.03	-0.12	-0.14	0.15	0.16	0.10	0.21	0.09	0.10	0.13	0.22	0.08	0.00	0.25	0.17	-0.09	0.11	-0.02	-0.24
**TrC38**	0.43	0.41	0.44	0.10	0.15	0.30	-0.05	-0.18	-0.22	-0.17	-0.17	-0.10	-0.23	-0.12	-0.20	-0.23	0.01	-0.13	-0.27	-0.24	-0.08	0.16	0.17	0.30
**TrC39**	-0.18	-0.18	-0.17	-0.17	-0.12	-0.14	0.08	0.06	0.04	0.00	0.12	0.20	0.05	-0.10	0.06	0.18	-0.06	0.11	0.07	0.26	0.09	-0.09	-0.19	-0.23

Supplementary Table 5 - Transcriptomic clusters Mann-Whitney p-values group comparison

	Days post boost				
**Cluster**	**0d**	**1d**	**3d**	**7d**	**14d**
**TrC01**	0.06	0.21	0.59	0.22	0.12
**TrC02**	0.07	0.26	0.59	0.23	0.12
**TrC03**	0.06	0.34	0.59	0.30	0.27
**TrC04**	0.06	0.08	0.59	0.45	0.27
**TrC05**	0.30	0.57	0.59	0.78	0.57
**TrC06**	0.04	0.11	0.59	0.28	0.24
**TrC07**	0.14	0.72	0.75	0.30	0.27
**TrC08**	0.06	0.04	0.59	0.30	0.35
**TrC09**	0.33	0.34	0.59	0.23	0.13
**TrC10**	0.73	0.43	0.59	0.30	0.14
**TrC11**	0.47	0.96	0.99	0.30	0.40
**TrC12**	0.06	0.51	0.66	0.22	0.27
**TrC13**	0.91	0.96	0.90	0.86	0.96
**TrC14**	0.04	0.34	0.59	0.23	0.27
**TrC15**	0.33	0.28	0.59	0.30	0.21
**TrC16**	0.96	0.51	0.59	0.66	0.39
**TrC17**	0.14	0.96	0.93	0.22	0.54
**TrC18**	0.47	0.34	0.76	0.84	0.93
**TrC19**	0.07	0.08	0.59	0.23	0.27
**TrC20**	0.53	0.96	0.65	0.66	0.27
**TrC21**	0.53	0.66	0.90	0.23	0.90
**TrC22**	0.33	0.38	0.59	0.85	0.99
**TrC23**	0.82	0.34	0.59	0.86	0.99
**TrC24**	0.33	0.21	0.59	0.30	0.21
**TrC25**	0.06	0.28	0.59	0.23	0.27
**TrC26**	0.64	0.34	0.59	0.38	0.99
**TrC27**	0.00	0.02	0.21	0.23	0.04
**TrC28**	0.47	0.72	0.66	0.85	0.82
**TrC29**	0.33	0.96	0.59	0.30	0.50
**TrC30**	0.91	0.56	0.59	0.58	0.21
**TrC31**	0.91	0.34	0.38	0.01	0.27
**TrC32**	0.15	0.57	0.66	0.22	0.13
**TrC33**	0.96	0.96	0.70	0.30	0.65
**TrC34**	0.06	0.90	0.90	0.84	0.73
**TrC35**	0.82	0.96	0.99	0.86	0.27
**TrC36**	0.81	0.96	0.99	0.30	0.40
**TrC37**	0.10	0.36	0.59	0.84	0.51
**TrC38**	0.54	0.36	0.38	0.01	0.04
**TrC39**	0.14	0.69	0.59	0.85	0.41

Supplementary Table 6 – Cohort demographic information

DONOR ID	AGE	GENDER	ETHNICITY	RACE	COHORT	PRIMING VACCINE	BOOSTER VACCINE	VACCINATION DAY	BIRTH YEAR
1655	29	F	Not Hispanic or Latino	White	wP	DTP	Tdap	09/12/2016	1/1/86
1687	33	F	Unknown	White	wP	DTP	Tdap	10/10/2016	1/1/83
1829	28	M	Not Hispanic or Latino	Asian	wP	DTP	Tdap	08/29/2016	1/1/88
1832	24	M	Not Hispanic or Latino	Asian	wP	DTP	Tdap	08/29/2016	1/1/91
2383	28	F	Not Hispanic or Latino	White	wP	DTP	Tdap	10/10/2016	1/1/88
2500	34	F	Hispanic or Latino	More than one race	wP	DTP	Tdap	11/07/2016	1/1/81
2685	19	M	Not Hispanic or Latino	Asian	aP	DTP	Tdap	07/25/2016	1/1/96
2686	34	F	Not Hispanic or Latino	Asian	wP	DTP	Tdap	07/25/2016	1/1/82
2687	30	F	Hispanic or Latino	Unknown	wP	DTP	Tdap	08/29/2016	1/1/86
2688	33	M	Not Hispanic or Latino	Asian	wP	DTP	Tdap	07/25/2016	1/1/82
2689	19	M	Not Hispanic or Latino	White	aP	DTP	Tdap	07/25/2016	1/1/97
2690	22	M	Not Hispanic or Latino	White	wP	DTP	Tdap	08/15/2016	1/1/93
2691	27	M	Not Hispanic or Latino	Asian	wP	DTP	Tdap	08/15/2016	1/1/89
2693	34	F	Hispanic or Latino	Unknown	wP	DTP	Tdap	07/25/2016	1/1/87
2696	35	F	Hispanic or Latino	White	wP	DTP	Tdap	09/12/2016	1/1/80
2698	19	F	Hispanic or Latino	Unknown	aP	DTaP	Tdap	08/29/2016	1/1/97
2699	21	M	Not Hispanic or Latino	Asian	wP	DTP	Tdap	09/26/2016	1/1/94
2702	35	F	Not Hispanic or Latino	White	wP	DTP	Tdap	08/29/2016	1/1/81
2703	33	M	Not Hispanic or Latino	White	wP	DTP	Tdap	08/29/2016	1/1/83
2704	31	F	Not Hispanic or Latino	White	wP	DTP	Tdap	08/29/2016	1/1/85
2706	25	F	Not Hispanic or Latino	White	wP	DTP	Tdap	09/26/2016	1/1/91
2707	24	F	Not Hispanic or Latino	Asian	wP	DTP	Tdap	09/13/2016	1/1/92
2719	26	F	Not Hispanic or Latino	Black or African American	wP	DTP	Tdap	09/13/2016	1/1/88
2726	33	F	Hispanic or Latino	Unknown	wP	DTP	Tdap	09/26/2016	1/1/83
2728	19	F	Not Hispanic or Latino	Asian	aP	DTP	Tdap	09/26/2016	1/1/97
2729	34	M	Unknown	Unknown	wP	DTP	Tdap	09/26/2016	1/1/82
2730	19	M	Hispanic or Latino	White	aP	DTaP	Tdap	09/26/2016	1/1/97
2731	27	F	Hispanic or Latino	White	wP	DTP	Tdap	09/26/2016	1/1/88
2732	26	F	Not Hispanic or Latino	Asian	wP	DTP	Tdap	09/26/2016	1/1/89
2736	19	M	Not Hispanic or Latino	Native Hawaiian or Other Pacific Islander	aP	DTaP	Tdap	10/24/2016	1/1/97
2738	26	M	Hispanic or Latino	More than one race	wP	DTP	Tdap	10/10/2016	1/1/90
2883	33	F	Hispanic or Latino	Unknown	wP	DTP	Tdap	10/24/2016	1/1/83
2884	25	M	Unknown	White	wP	DTP	Tdap	10/10/2016	1/1/91
2886	18	F	Hispanic or Latino	White	aP	DTaP	Tdap	10/24/2016	1/1/97
2887	18	F	Not Hispanic or Latino	More than one race	aP	DTaP	Tdap	11/07/2016	1/1/98
2890	19	F	Not Hispanic or Latino	White	aP	DTaP	Tdap	10/24/2016	1/1/97
2893	30	F	Not Hispanic or Latino	White	wP	DTP	Tdap	10/24/2016	1/1/85
2895	22	F	Not Hispanic or Latino	Asian	wP	DTP	Tdap	10/24/2016	1/1/94
2899	31	M	Not Hispanic or Latino	White	wP	DTP	Tdap	11/07/2016	1/1/85
2900	19	F	Not Hispanic or Latino	Asian	aP	DTaP	Tdap	11/07/2016	1/1/97
2901	18	F	Not Hispanic or Latino	More than one race	aP	DTaP	Tdap	11/07/2016	1/1/98
2902	18	F	Hispanic or Latino	More than one race	aP	DTaP	Tdap	11/07/2016	1/1/98
2903	19	F	Not Hispanic or Latino	Asian	aP	DTaP	Tdap	11/28/2016	1/1/97
2904	18	F	Not Hispanic or Latino	Unknown	aP	DTaP	Tdap	11/07/2016	1/1/98
2912	19	F	Not Hispanic or Latino	White	aP	DTaP	Tdap	11/28/2016	1/1/96
2915	18	F	Not Hispanic or Latino	White	aP	DTaP	Tdap	01/17/2017	1/1/98
2921	18	F	Not Hispanic or Latino	White	aP	DTaP	Tdap	01/17/2017	1/1/97
2922	19	F	Not Hispanic or Latino	Asian	aP	DTaP	Tdap	11/28/2016	1/1/97
2923	19	M	Not Hispanic or Latino	White	aP	DTaP	Tdap	11/28/2016	1/1/97
2928	18	M	Not Hispanic or Latino	More than one race	aP	DTaP	Tdap	01/03/2017	1/1/98
2935	18	F	Hispanic or Latino	Unknown	aP	DTaP	Tdap	01/03/2017	1/1/98
2952	19	F	Not Hispanic or Latino	Asian	aP	DTaP	Tdap	01/17/2017	1/1/97
2968	19	F	Not Hispanic or Latino	Asian	aP	DTaP	Tdap	01/17/2017	1/1/97
2976	19	F	Not Hispanic or Latino	Asian	aP	DTaP	Tdap	01/30/2017	1/1/97
2995	20	F	Not Hispanic or Latino	Asian	aP	DTaP	Tdap	01/30/2017	1/1/96
2997	19	F	Hispanic or Latino	Unknown	aP	DTaP	Tdap	01/30/2017	1/1/97
2998	19	F	Hispanic or Latino	More than one race	aP	DTaP	Tdap	01/30/2017	1/1/97
3000	19	M	Hispanic or Latino	White	aP	DTaP	Tdap	01/30/2017	1/1/97

Supplementary Table 7 – Proteomics excluded assays and exclusion criteria Olink PEA

Count	Olink panel	Assay	Uniprot ID	Reason for exclusion
1	Olink IMMUNE RESPONSE(v.3202)	ARNT	P27540	≤ LOD
2	Olink IMMUNE RESPONSE(v.3202)	BIRC2	Q13490	≤ LOD
3	Olink IMMUNE RESPONSE(v.3202)	DGKZ	Q13574	≤ LOD
4	Olink IMMUNE RESPONSE(v.3202)	EIF5A	P63241	≤ LOD
5	Olink METABOLISM(v.3402)	GLRX	P35754	≤ LOD
6	Olink IMMUNO-ONCOLOGY(v.3101)	IFN-beta	P01574	≤ LOD
7	Olink IMMUNO-ONCOLOGY(v.3101)	IFN-gamma	P01579	≤ LOD
8	Olink IMMUNE RESPONSE(v.3202)	IFNLR1	Q8IU57	≤ LOD
9	Olink IMMUNO-ONCOLOGY(v.3101)	IL-1 alpha	P01583	≤ LOD
10	Olink IMMUNO-ONCOLOGY(v.3101)	IL-21	Q9HBE4	≤ LOD
11	Olink IMMUNO-ONCOLOGY(v.3101)	IL-35	Q14213,P29459	≤ LOD
12	Olink IMMUNO-ONCOLOGY(v.3101)	IL13	P35225	≤ LOD
13	Olink IMMUNO-ONCOLOGY(v.3101)	IL2	P60568	≤ LOD
14	Olink IMMUNO-ONCOLOGY(v.3101)	IL33	O95760	≤ LOD
15	Olink IMMUNE RESPONSE(v.3202)	IRAK4	Q9NWZ3	≤ LOD
16	Olink IMMUNE RESPONSE(v.3202)	KPNA1	P52294	≤ LOD
17	Olink IMMUNE RESPONSE(v.3202)	NF2	P35240	≤ LOD
18	Olink METABOLISM(v.3402)	NQO2	P16083	≤ LOD
19	Olink IMMUNO-ONCOLOGY(v.3101)	PTN	P21246	≤ LOD
20	Olink METABOLISM(v.3402)	S100P	P25815	≤ LOD
21	Olink IMMUNO-ONCOLOGY(v.3101)	TNF	P01375	≤ LOD
22	Olink IMMUNE RESPONSE(v.3202)	CD28	P10747	≤ LOD
23	Olink IMMUNO-ONCOLOGY(v.3101)	CD28	P10747	≤ LOD
24	Olink IMMUNO-ONCOLOGY(v.3101)	CD83	Q01151	Duplicate
25	Olink IMMUNO-ONCOLOGY(v.3101)	CXCL12	P48061	Duplicate
26	Olink IMMUNO-ONCOLOGY(v.3101)	FGF2	P09038	Duplicate
27	Olink IMMUNO-ONCOLOGY(v.3101)	IL10	P22301	Duplicate
28	Olink IMMUNO-ONCOLOGY(v.3101)	IL12RB1	P42701	Duplicate
29	Olink IMMUNO-ONCOLOGY(v.3101)	IL5	P05113	Duplicate
30	Olink IMMUNO-ONCOLOGY(v.3101)	IL6	P05231	Duplicate
31	Olink IMMUNO-ONCOLOGY(v.3101)	KLRD1	Q13241	Duplicate
32	Olink IMMUNO-ONCOLOGY(v.3101)	LAMP3	Q9UQV4	Duplicate
33	Olink IMMUNO-ONCOLOGY(v.3101)	NCR1	O76036	Duplicate
34	Olink IMMUNO-ONCOLOGY(v.3101)	ANGPT2	O15123	Duplicate
35	Olink IMMUNO-ONCOLOGY(v.3101)	ARG1	P05089	Duplicate

Supplementary Table 8 – CyTOF antibodies and conjugations

Marker	Metal	Clone	Cat#	Source
CD45	89Y	HI30	3089003B	Fluidigm
CD3	115In	UCHT1	300443	Biolegend
CD19	142Nd	HIB19	3142001B	Fluidigm
CD38	144Nd	HIT2	3144014B	Fluidigm
CD4	145Nd	RPA-T4	3145001B	Fluidigm
CD20	145Nd	2H7	302302	Biolegend
CD123	151Eu	6H6	3151001B	Fluidigm
CD45RA	155Gd	HI100	3155011B	Fluidigm
CD1c	160Gd	L161	331502	Biolegend
CD33	163Dy	WM53	3163023B	Fluidigm
CCR7	167Er	G043H7	3167009A	Fluidigm
CD25	169Tm	M-A251	3169003B	Fluidigm
CD8a	172Yb	RPA-T8	301002	Biolegend
CD14	173Yb	61D3	14-0149-82	Thermo Fisher Scientific
HLA-DR	174Yb	L243	3174001B	Fluidigm
CD56	176Yb	CMSSB	3176003B	Fluidigm
CD16	209Bi	3G8	3209002B	Fluidigm

Supplementary Methods

RNA-seq analysis

Clustering analysis. For clustering RNA-Seq data, weighted gene co-expression network analysis (WGCNA) was performed to identify sets of genes that share a similar expression (Langfelder et al. 2008, Langfelder et al. 2012) WGCNA parameters were set in the following way: minModuleSize = 20, deepSplit = 4 and MEDissThres= 0. The 8,000 most variable genes that were considerably expressed (max (TMP > 5) among all samples) were considered as input for the WGCNA analysis.

For clustering of proteomics data (PrCXX clusters), only proteins with average intra-donor coefficient of variation greater than 0.1 were considered, i.e. those which were substantially altered after the immunization. Then the distance matrix across all samples using correlation metric was computed. 'Average' linkage method was applied to construct dendrograms. Threshold for cluster assignment was chosen based on similar approach as for transcriptomic data, finding a trade-off between inclusion of proteins into clusters and cluster tightness (**Supplementary Figure 5**). Detailed code used for RNA-Seq and proteomic cluster analysis can be found in https://github.com/JasonPBennett/Pertussis_2020 for reproducing the results.

Identification of perturbed clusters. For each of the transcriptomic modules or proteomic clusters, the first principal component was extracted by collapsing gene/protein dimension. Then for each of the modules/clusters, two timepoints were identified: the timepoint with the highest PC1 value and the timepoint with the lowest PC1 value averaging using the median across the donors considered. Then Wilcoxon test (paired) was applied to the highest point vs. lowest point to check statistical significance of the perturbations among the two timepoints for a particular module/cluster. Obtained p-values were then FDR corrected for the number of modules/clusters considered.

Identification of common longitudinal perturbation patterns among RNA-Seq clusters. PC1 of every cluster was mean centered and min-max normalized. These normalized PC1 values varying from -1.0 to 1.0 (due to min-max normalization) were subject to hierarchical clustering. For computation of pairwise distance, *scipy.spatial.distance.pdist* function with metric "cityblock" was used. Distance matrix was linked using *scipy.cluster.hierarchy.linkage* function with "average" method. Resulting dendrogram was cut at 0.4 of maximum variance. Resulting group clusters are shown in **Supplementary Figure 2**.

Differential expression analysis. The analysis was done using Bioconductor package DESeq2 (Banus et al. 2006). For each of the timepoints profiled, aP vs. wP analysis was undertaken. To reduce the number of comparisons, only the genes with mean TPM>1 across all the donors for a particular timepoint were taken into consideration. Genes were considered differentially expressed when the analysis resulted in a Benjamini-Hochberg $p_{adj} < 0.05$.

Function characterization of gene sets. For inferring transcriptional patterns of genes belonging to a cluster, the first principal component was extracted using principal component analysis reducing the gene/protein dimension. Gene Ontology enrichment was done in GOnet web application (Subramanian et al. 2005).

Statistical comparisons of donor groups. To find gene modules significantly different between aP and wP individuals at different timepoints two-sided Mann Whitney U-test with continuity correction was used to reject null hypothesis of the same PC1 value of the module considered. To control for multiple comparisons due to multiple gene modules and timepoints, FDR correction Benjamini-Hochberg was used. Perturbation of gene expression and cell type frequencies were evaluated using Paired Wilcoxon test. Comparisons between groups at determinate timepoints were made using the nonparametric Two-tailed Unpaired Mann-Whitney U test. Prism 5.0 (GraphPad) was used for all these

calculations. All data in all figures in which error bars are shown is presented as median ± interquartile range. A p value <0.05 was considered statistically significant and specific p values are shown for each figure.
